# High-throughput behavioural phenotyping of 25 *C. elegans* disease models including patient-specific mutations

**DOI:** 10.1186/s12915-025-02368-8

**Published:** 2025-09-26

**Authors:** Thomas J. O’Brien, Eneko P. Navarro, Consuelo Barroso, Lara Menzies, Enrique Martinez-Perez, David Carling, André E. X. Brown

**Affiliations:** 1 School of Molecular and Cellular Biology, University of Leeds, Leeds, UK; 2 MRC Laboratory of Medical Sciences, London, UK; 3 Institute of Clinical Sciences, Imperial College London, London, UK; 4 Department of Clinical Genetics, Great Ormond Street Hospital for Children, NHS Foundation Trust, London, UK

**Keywords:** Caenorhabditis elegans, High-throughput phenotyping, Multidimensional behaviour, Disease modelling, Patient avatar, Allelic variant

## Abstract

**Background:**

Genetic diagnosis is fast and cheap, challenging our capacity to evaluate the functional impact of novel disease-causing variants or identify potential therapeutics. Model organisms including *C. elegans* present the possibility of systematically modelling genetic diseases, yet robust, high‐throughput methods have been lacking.

**Results:**

Here we show that automated multi‐dimensional behaviour tracking can detect phenotypes in 25 new *C. elegans* disease models spanning homozygous loss‐of‐function alleles and patient‐specific single‐amino‐acid substitutions. We find that homozygous loss‐of‐function (LoF) mutants across diverse genetic pathways (including BORC, FLCN, and FNIP‐2) exhibit strong, readily detectable abnormalities in posture, locomotion, and stimulus responses compared to wild‐type animals. An *smc-3* mutant strain—modelled by introducing a patient‐identified missense change—exhibited developmental anomalies and distinct behavioural profiles even though complete loss of SMC‐3 is lethal. In contrast, patient-derived missense mutations in another essential gene, *tnpo-2*, did not show a strong phenotype initially but it could be “sensitized” chemically (e.g., with aldicarb), potentially facilitating future drug screens.

**Conclusions:**

Our findings show that scalable behavioural phenotyping can capture a wide range of mutant effects—from strong to subtle—in patient‐avatar worm lines. We anticipate that this standardized approach will enable systematic drug repurposing for rare genetic disorders as new disease variants are discovered.

**Supplementary Information:**

The online version contains supplementary material available at 10.1186/s12915-025-02368-8.

## Background

Advances in genome and exome sequencing technologies mean the rates of genetic variant classification and drug development cannot keep pace with the rate of disease gene discovery. More than 100 genes associated with new genetic disorders are discovered and uploaded to databases such as OMIM [1, 2] every year. Genetic diagnosis of disease is an important first step, but identifying the genetic cause associated with the onset of a disorder does not necessarily lead to a drug target hypothesis. This is particularly true for rare diseases which are often poorly characterised and underfunded: ~ 95% have no approved treatment or validated therapeutic target [2]. Approximately 74% of rare diseases affect the central nervous system [3], giving rise to pleiotropic presentations of disease in both the clinic and in vivo vertebrate models, which complicates investigation into disease onset and progression [4, 5]. Moreover, different variants within the same gene can result in different phenotypes [6]. There is therefore a need to develop methods to efficiently study patient-specific mutations to support the development of personalised therapies.

In the absence of a validated drug target, phenotypic screens in whole organism models provide an alternative route for drug discovery. However, the use of in vivo models in high-throughput studies can be hampered by the lack of a robust and rapidly detected phenotype. The cost and speed of making and validating new genetic and phenotyping methods for new disease/allelic variants is a further challenge.

We have recently shown that high-throughput tracking shows promise for systematic phenotyping and drug repurposing using a single multiwell plate assay [7]. Using *C. elegans* as a model organism, we followed a disease-phenolog approach to develop a standardised screening method that combines high-resolution worm tracking [8] with automated quantitative behavioural phenotyping [7, 9–14]. Because we detect multiple phenotypes with a single assay, multiple mutations or treatment conditions can be screened using the same approach without prior knowledge of the molecular underpinnings of a disease other than the mutation to model.

Previously we focussed on diseases that could be modelled using knockouts. Here we extend the approach to characterise 25 additional *C. elegans* disease models encompassing homozygous loss-of-function (LoF), heterozygous LoF, and single amino acid substitution mutants. We detected behavioural differences for all strains compared to wild-type. The mutations affect diverse genetic pathways and we detected correspondingly diverse phenotypes. Despite this diversity, we find that mutations in genes that are involved in related cellular processes lead to similar phenotypic profiles.

The multidimensional behavioural fingerprinting of *C. elegans* patient avatars provides a foundation for scalable drug discovery efforts using a readily-perturbable whole organism model. The systematic creation, phenotyping, and screening of patient-specific genetic variants may form the basis for the discovery of lead compounds and the development of personalised therapeutics at a rate commensurate with disease gene discovery.

## Results

### Behavioural phenotypes are detected for all disease model mutants

The 25 mutant strains made for this study are primarily based on genetic variants found in patients that were identified by collaborators and they cover 25 different genes associated with a diverse representation of rare genetic disorders. Different strategies were used depending on the specific mutation and its phenotype. All mutants were initially characterised using a previously developed pipeline [7, 8, 11–14]. Briefly, young adult worms are added to the wells of a 96-well plate using a COPAS Biosort and recorded on custom imaging rigs (LoopBio). We record a 16-min video comprising: (1) a 5-min pre-stimulus recording (capturing differences in baseline behaviour), (2) a 6-min blue light recording (testing differences in a strains’ photophobic escape response [15]), (3) a 5-min post-stimulus recording (testing recovery to stimulation). We then use Tierpsy [9] to extract 2763 features (covering morphology, posture and locomotion) for each video which are concatenated to make a phenotypic profile which is averaged over the worms in each well (8289 features in total) [10, 16].

**Fig. 1:**
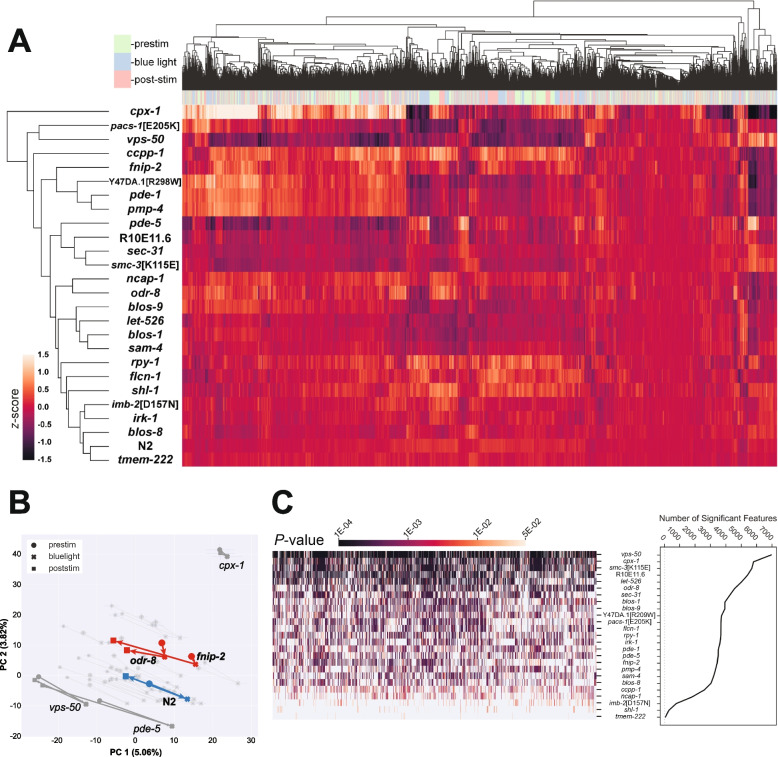
All disease model mutants exhibit behavioural differences compared to the control. **A** Hierarchical clustering of the entire behavioural feature set extracted by Tierpsy (8289 features total). Features are *Z*-normalised. The top barcode shows when during the period of image acquisition the behavioural features were extracted: pre-stimulation (pink), blue light (blue) and post-stimulation (green). **B** Principal component analysis of the disease model mutants and wild-type reference (blue). Strains move in phenospace between pre-stimulus (circular points), blue light (crosses) and post-stimulation (squares) recordings. Mutant strains that do not return towards their pre-stimulus position in phenomic space are coloured red. **C** The total number of statistically significant behavioural features extracted for each strain compared to N2. *p*-values for each feature are calculated using block permutation t-tests, using *n* = 100,000 permutations and *p* < 0.05 is considered statistically significant after correcting for multiple comparisons using the Benjamini-Yekutieli method. For each strain, data represent *n* ≥ 135 wells, each containing 3–5 worms and contributing a single averaged behavioural measurement

Hierarchical clustering of the different patient avatars reveals that different behavioural phenotypes are captured across this diverse panel of mutant strains (Fig. 1A). Phenotypic similarities are also captured for strains that have mutations within genes predicted to have a similar function or which are associated with similar diseases in humans. For example, *blos-1*, *blos-9*, and *sam-4* are all predicted to be members of the BLOC-one-related complex (BORC) [17]. LoF mutations within these genes result in very similar behavioural phenotypes. However, this is not always the case, as our *blos-8* LoF mutant (also predicted to be a subunit of the BORCS complex) has a markedly different behavioural phenotype.

We next performed principal component analysis (PCA) on the same data, but separated into the pre-stimulus, blue light, and post-stimulus tracking (Fig. 1B). Blue light stimulation affects worm behaviour which corresponds to a shift of points in phenotype space. Most strains show a partial phenotypic recovery (similar to wild-type N2) during post-stimulation recordings. However, *odr-8*(*syb4940*) and *fnip-2*(*syb8038*) mutants show a reduced recovery (Fig. 1B).

Of the 25 disease model strains, 22 had a ‘strong’ behavioural phenotype, exhibiting > 1000 statistically significant behavioural differences compared to N2 (Fig. 1C). These strains are more likely to be suitable for drug screens where many compounds are tested, which would typically necessitate a smaller number of replicates. It is important to note that not all the phenotypes extracted by Tierpsy are independent and some features are partially correlated. For example, speed features measured for different body parts are usually related. Therefore, a worm moving uniformly slower than N2 will show a significant reduction across hundreds of speed-related features. Thus, the total number of significant behavioural differences within the feature set is a rough proxy for effect size as the result of a genetic mutation. This is further illustrated in Fig. 1C, where strains with the largest number of statistically significant differences also tend to show lower *p*-values (darker lines), reflecting stronger overall phenotypic effects.

Strain-specific phenotype summaries, the molecular characteristics, previously reported phenotypes (if available) and human disease(s) associated with each strain are available in Additional File 1 (strain-specific gene cards) [6, 18–47]. We have also created an interactive heatmap of the hierarchical cluster map (Fig. 1A) that can be used to explore the behavioural differences of all strains compared to wild-type (available at https://zenodo.org/records/13941390).

### BORC complex mutations

BORC is a multi-subunit protein complex that enables the correct positioning of lysosomes within cells [17]. By promoting the recruitment of the small GTPase ARL8 [48], which in-turn recruits the motor proteins kinesin-1 [49] and kinesin-3 [50], BORC enables the anterograde (centrifugal) movement of lysosomes along microtubule tracks in non-polarised cells [17, 49, 51] and enables movement towards the distal axon tip in neurons [52–54]. Truncation of any member of the complex results in the juxtanuclear positioning of lysosomes [55]. Furthermore, genetic variants within BORC genes are associated with many neurodegenerative disorders [56] including: Hermansky-Pudlak Syndrome [57], hereditary spastic paraplegia [54, 58, 59], Parkinson’s Disease [60, 61], Huntingdon’s Disease [62], Alzheimer’s [63], amyotrophic lateral sclerosis [64], schizophrenia [65, 66], and neuronal axonal dystrophy [67]. Despite its widespread expression, the consequences of BORC deficiency primarily manifest in the nervous system. Thus, BORC is an emerging research target for the treatment of many neurodevelopmental conditions [60, 68].

BORC is conserved between vertebrate [6], invertebrate [41, 50, 69, 70] and prokaryotic [71] species. *C. elegans* embryos have been used to study defective phagolysosome clearance in *sam-4* (*BORCS5* ortholog) and *blos-7* (*BORCS6* ortholog) LoF mutants [70], and *borcs8* knockout (*BORCS8* ortholog) zebrafish are found to have general neurodevelopmental delay and locomotive defects [6]. Here we selected 4 genes associated with BORC: *blos-1* (*BLOCS1* ortholog), *blos-8* (*BORCS7* ortholog), *blos-9* (*BORCS9* ortholog), and *sam-4* and created homozygous deletions to model BORC deficiency.

**Fig. 2:**
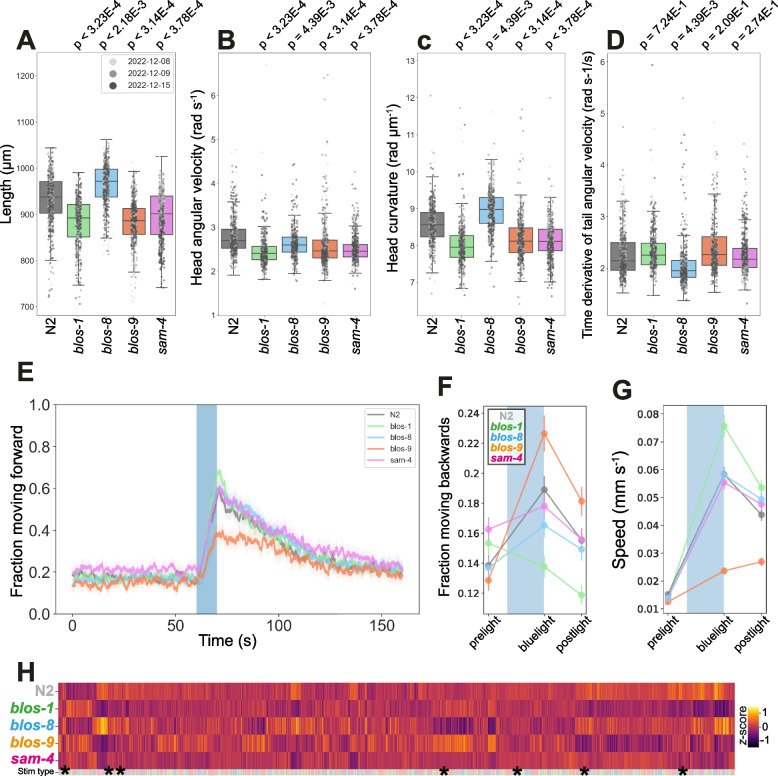
BORC deficiency disease model phenologs. **A**-**D** Key behavioural features altered in strains containing loss-of-function mutations in orthologs associated with BORC: *blos-1*(*syb6895*), *blos-8*(*syb6686*), *blos-9*(*syb7029*) and *sam-4*(*syb6765*). Individual points marked on the box plots are well averaged values (3 – 5 worms per well) for each feature across the independent days of tracking. *p*-values are for comparison to wild-type N2 using block permutation t-tests (*n* = 100,000 permutations, correcting for multiple comparisons using the Benjamini-Yekutieli method). **E** Overall fraction of worms moving forward 60 s prior to and 80 s following stimulation with a 10 s blue light pulse (blue shading). Coloured lines represent averages of the detected fraction of paused worms across all biological replicates and shaded areas represent 95% confidence intervals. **F**-**G** Changes in selected features in response to stimulation with a single 10 s pulse of blue light (shaded region). Feature values are calculated using 10 s windows centred on 5 s before, 10 s after, and 20 s after the beginning of the pulse. **H** Heatmap of the entire set of 8289 behavioural features extracted by Tierpsy for the disease model strains associated with BORC deficiency. The ‘stim type’ barcode denotes when during image acquisition a feature was extracted: pre-stimulation (pink), blue light stimulation (blue) and post-stimulation (green). Asterisks show the location of the selected features presented in A-D. Each strain is represented by data from *n* > 495 well averaged values

Mutants with homozygous deletions of these BORC genes are viable (in contrast to vertebrates [6, 52, 54]) and show strong behavioural phenotypes with >3000 features significantly different from N2. *blos-1*(*syb6895*), *blos-9*(*syb7029*) and *sam-4*(*syb6765*) are all shorter, whereas *blos-8*(*syb6686*) is longer, than N2 (Fig. 2A). Furthermore, *blos-1*, *blos-9*, and *sam-4* LoF primarily affects head-related features, including decreased angular velocity (Fig. 2B), curvature (Fig. 2C), and acceleration or speed of the head (strain-specific gene cards, Additional File 1). In contrast, *blos-8* LoF displayed a significant increase in head curvature (Fig. 2C) and affected other areas of the worm body, for example resulting in a decrease in tail angular acceleration (Fig. 2D). Such confounding phenotypes are consistent with reports that BLOS-8 and KXD-1 (encoded by *C. elegans kxd-*2) are the only members of the BORCS complex that are dispensable for synaptic vesicle transport but are essential for lysosomal transport [72]. Additionally, truncation of mouse *Borcs7* (*blos-8* ortholog) causes axon degradation and severe motor defects in mice [54], suggesting that *blos-8*’s role in contributing towards neuronal health and motor function is evolutionarily conserved.

We detect no differences in the fraction of time worms spend moving forwards between wild-type (N2) worms and *blos-8*(*syb6686*) or *sam-4*(*syb6765*), but *blos-1*(*syb6895*) has an increased forward and decreased backward response to stimulation with blue light (Fig. 2E-G). In contrast, *blos-9*(*syb7029*) has a decreased forward and increased backward response. Loss of BLOS-9 disrupts the ARL-8/UNC-104 anterograde transport pathway in worms [72]. This is likely to reduce synaptic vesicle delivery and neurotransmission, thereby dampening locomotion in response to an external stimulus. We are unsure as to why *blos-1* LoF would result in a different phenotype. Given that *blos-1* is the only member of the BORCS complex known to also interact with the BLOC-1complex (mediating biogenesis of lysosome-related organelles) it is possible that disruption of cellular metabolism because of dysregulated BLOC-1 function influences synaptic function, and by extension locomotion.

### Folliculin mutations

Birt-Hogg-Dubé syndrome (BHD) is a rare autosomal dominant disorder characterised by fibrofolliculomas, lung cysts, spontaneous pneumothorax and renal cell carcinomas [73, 74]. Variants in the *FLCN* gene are found to be responsible for BHD [73]. FLCN is highly conserved from unicellular organisms to mammals [75]. Studies in mice identified two conserved binding partners of FLCN, FNIP1 and FNIP2, that regulate AMPK (5′-AMP activated protein kinase) activation [76, 77]. Despite this known interaction, their precise role is unclear as both inhibition and stimulation of AMPK have been reported [75]. Recently an association of FNIP1/FINP2 in glucose homeostasis has been confirmed using a mouse model with an adipose tissue specific ablation of FNIP1 [78]. FLCN is also reported to have a role in mTOR suppression [79], mTORC regulation [80–83], ciliogenesis [84], transforming growth factor-b signalling [85, 86], autophagy [87–89], cell adhesion [83], cell polarity [90], and regulation of the cell cycle [91, 92].

**Fig. 3:**
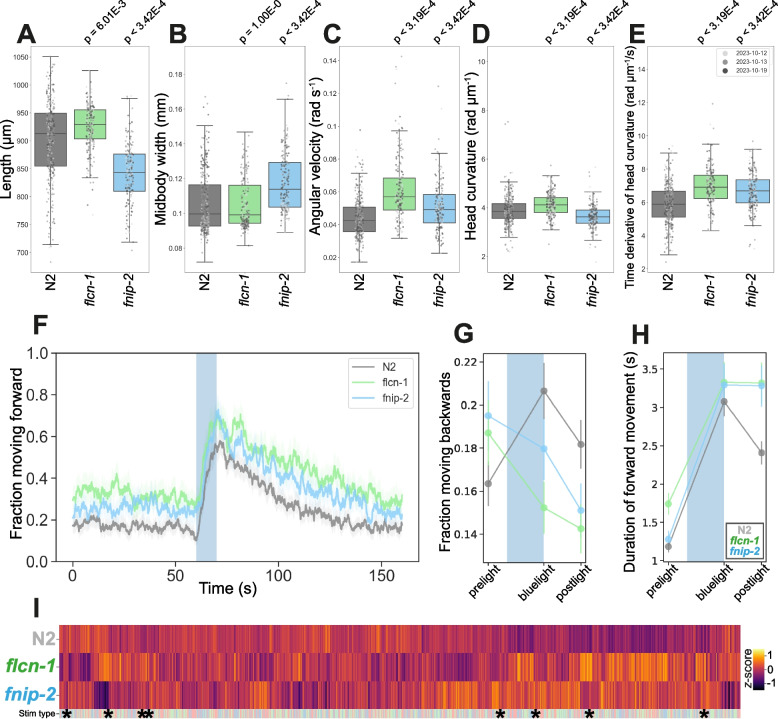
Folliculin mutant disease model phenologs. **A**-**E** Key behavioural features altered in *s*trains containing loss-of-function mutations in the *C. elegans* orthologs of *FLCN*, *flcn-1*(*syb8071*), or *FNIP1/2*, *fnip-2*(*syb8038*). Individual points marked on the box plots are well averaged values (3 – 5 worms per well) for each feature across the independent days of tracking. *p*-values are for comparison to wild-type N2 using block permutation t-tests (*n* = 100,000 permutations, correcting for multiple comparisons using the Benjamini-Yekutieli method). **F** Overall fraction of worms moving forward 60 s prior to and 80 s following stimulation with a 10 s blue light pulse (blue shading). Coloured lines represent averages of the detected fraction of paused worms across all biological replicates and shaded areas represent 95% confidence intervals. **G**-**H** Changes in selected features in response to stimulation with a single 10 s pulse of blue light (shaded region). Feature values are calculated using 10 s windows centred on 5 s before, 10 s after, and 20 s after the beginning of the pulse. **I** Heatmap of the entire set of 8289 behavioural features extracted by Tierpsy for our *flcn-1* and *fnip-2* LoF mutants. The ‘stim type’ barcode denotes when during image acquisition a feature was extracted: pre-stimulation (pink), blue light stimulation (blue) and post-stimulation (green). Asterisks show the location of the selected features presented in A-E. Data shown represents *n* > 220 well averaged values for all strains. Each strain is represented by data from *n* > 229 well averaged values

Homozygous deletion of *flcn-1* (FLCN ortholog) or *fnip-2* (FNIP1 and FNIP2 ortholog) in *C. elegans* is non-lethal, exhibits no developmental delay and results in strong behavioural phenotypes (3663 or 3433 statistically significant differences, respectively, compared to N2) (Fig. 3). *flcn-1*(*syb8071*) is longer than N2 with no difference in width (Fig. 3A-B). Consistent with reports that increased expression of FNIP2 in humans results in a decrease in body mass index [93], *fnip-2*(*syb8038*) loss-of-function mutants are shorter and wider than wild-type. Knock out of *flcn-1*(*syb8071*) may shift worm metabolism to a catabolic (low nutrient) program through the chronic activation of AMPK [87]. This is consistent with a normal width but does not immediately explain the longer length of these worms.

Both *flcn-1*(*syb8071*) and fnip*−2*(*syb8038*) have increased angular velocity for all body segments (Fig. 3C) and increased time derivative of curvature (Fig. 3E and Additional File 1). Deletion of *flcn-1* displays a significant increase in head curvature whereas deletion of *fnip-2* displays a decrease in this phenotype (Fig. 3D). Both mutants are hyperactive compared to the control (Fig. 3F), with a decreased backward blue light response (Fig. 3G) and more sustained forward blue light response (Fig. 3H).

Given the role of FLCN and FNIP1/2 in the regulation of AMPK and mTOR signalling, we tested a panel of compounds known to modulate AMPK/mTOR pathways to determine how they altered the behaviour of *flcn-1*(*syb8071*), *fnip-2*(*syb8038*) or N2. Due to the known antimicrobial activity of rapamycin (an mTOR inhibitor), we used PFA-killed OP50 as a food source for these experiments [94].

**Fig. 4:**
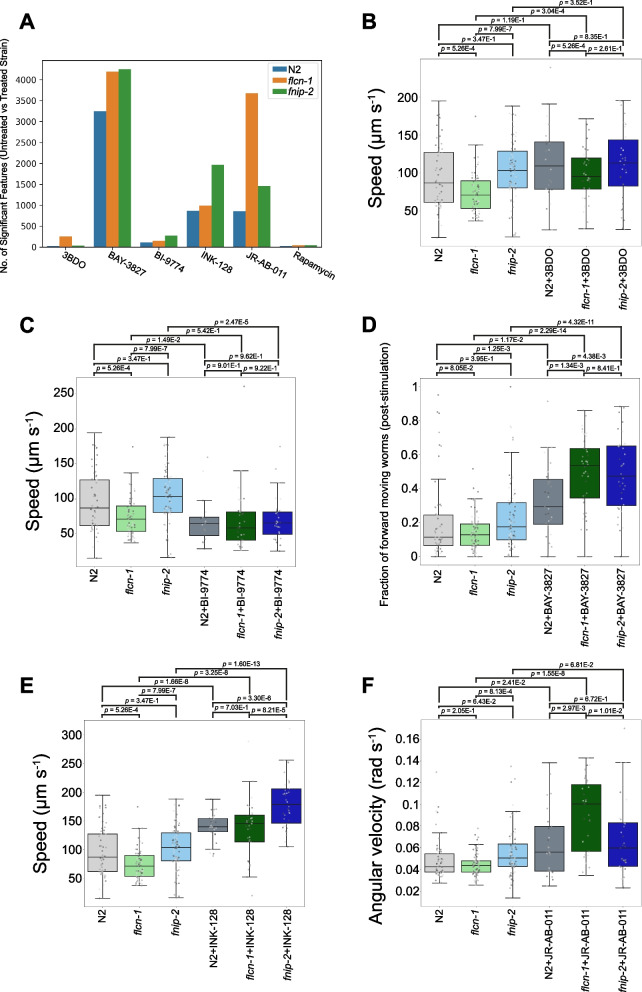
Effect of AMPK and mTOR inhibition or activation on folliculin mutant phenotypes. **A** Total number of behavioural changes detected between either N2, *flcn-1*(*syb8071*) or *fnip-2*(*syb8038*) treated with a compound vs the same strain treated with DMSO (untreated). Worms were exposed to 100 µM of each compound for 4 h prior to tracking. Statistically significant phenotypic changes were calculated using the Kruskal–Wallis test correcting for multiple comparisons using the Benjamini-Yekutieli method. **B**-**F** Key behavioural features differing between untreated strains and worms treated with the various AMPK and mTOR activator or inhibitors. Individual points marked on the box plots are well averaged values (3 – 5 worms per well) for each feature across the independent days of tracking. For all conditions, data represents *n* > 17 wells

Pairwise comparison of feature vectors extracted from strains exposed to DMSO only (untreated) vs 4 h treatment with 100 µM of each compound revealed no discernible change in the behaviour of any strain treated with rapamycin (Fig. 4A). Treatment with 3BDO (mTOR activator) has no effect on N2 or *fnip-2*(*syb8038*), but leads to 249 significant feature differences between untreated and treated *flcn-1*(*syb8071*) mutants (Fig. 4A-B). Although this is a weak phenotype, there is a consistent change in some speed-related features, alongside rescue of the decreased speed phenotype we observed between untreated N2 and the untreated *flcn-1* LoF strain (Fig. 4B).

Treatment with the pan-AMPK activator, BI-9774, results in a significant decrease in the speed of N2 and *fnip-2*(*syb9038*). There is no statistically significant difference in the speed of any of the treated strains after 4 h exposure to this compound (Fig. 4C). Although the phenotypic changes observed in *fnip-2*(*syb8038*) as a result of BI-9774 treatment are weak (only 337 significant differences), the differences are consistent with an increased susceptibility to AMPK activation in mutants lacking FNIP1/2. In contrast, treating worms with BAY-3827 (selective AMPK inhibitor) displays a very strong change in the behaviour of the wild-type and mutant strains (Fig. 4A), primarily an increase in most speed-related features. There are > 1000 significant phenotypic changes detected between the BAY-3827 treated and untreated mutants compared to the number of phenotype changes detected between untreated N2 and N2 treated with the BAY-3827 (Fig. 4A), suggesting that deletion of *flcn-1* or *fnip-2* makes worms more susceptible to AMPK inhibition. As an example, both mutants exhibit a greater fraction of the population moving after the cessation of blue light stimulation (Fig. 4D).

Like AMPK inhibition, treatment with INK-128 (an mTOR1/2 inhibitor) primarily led to an increase in speed-related features across all the strains (Fig. 4E). Exposure to INK-128 results in a > twofold increase in the number of behavioural differences detected between the treated and untreated *fnip-2* LoF worms (1969 feature differences) compared to *flcn-1*(*syb8071*) (994 features) or N2 (873 features, Fig. 4A). Furthermore, the increase in speed following mTOR1/2 inhibition is more pronounced for the *fnip-2* LoF mutant compared to the other strains (Fig. 4E). Treatment with the selective mTORC2 inhibitor, JR-AB-011, displayed a similar increase in the speed and locomotive-related features for all strains and we note little difference in the total number of phenotypic changes between N2 treated with INK-128 or JR-AB-011 (Fig. 4A). The *flcn-1* LoF mutant is more susceptible to treatment with JR-AB-011 and > 2000 more significant phenotypic differences are observed for treated *flcn-1*(*syb8071*) compared to *fnip-2*(*syb8038*) or wild-type. For example, we observe a strong increase in the angular velocity of the *flcn-1* LoF mutant upon JR-AB-011 treatment that is not observed in the other strains (Fig. 4F). Inhibition of mTOR1/2 results in more pronounced phenotypic changes (increased susceptibility) for worms lacking the FNIP1/2 ortholog, but knock-out of the FLCN ortholog results in a greater susceptibility of worms to mTORC2 inhibition.

PCA analysis of the phenotypes of the strains treated with the different AMPK and mTOR inhibitor/activators reveals that none of the treatments tested restored the mutant phenotypes to wild-type (Additional File 2). However, treatment with the different compounds moves both the mutant and wild-type strains in the same direction within phenotypic space.

We also attempted to use Western Blotting to validate the molecular underpinnings of these phenotypic changes as a result of differences in AMPK and mTOR signalling. For this we tried to measure the phosphorylation of T172 in AMPKa or S49 (worm position of human S51) eIF2a as a measure of AMPK [95] or mTORC1 [96, 97] signalling, respectively. Although we observed differences in the basal level of signalling between the untreated strains, we could not reliably identify changes in the phosphorylation state of either site following treatment with the different drugs (Additional File 3). These results could be due to a lack of specificity of the available antibodies to worm AMPKa and eIF2a phosphorylation sites or the transient nature of the phosphorylation states.

### Enhancing the separation of a TNPO2 patient avatar in phenomic space

The behavioural phenotypes we describe above relate to strains containing CRISPR deletions of an entire gene coding region (putative LoF mutants). However, some pathological mutations do not cause complete loss of function so a knockout may not be the best model. We created several strains with patient-specific single amino acid changes: *imb-2[D157N]* (ortholog of human *TNPO2[D156N]*), *pacs-1[E205K]* (ortholog of human *PACS2[E209K]*), *smc-3[K115E]* (ortholog of human *SMC3[K114E]*), and *Y47D91A.1[R298W]* (ortholog of human *GMPPA[R318W]*).

The *pacs-1[E205K]*, *smc-3[K115E]* (described in detail in the next section), and *Y47D91A.1[R298W]* mutants exhibit strong behavioural phenotypes (> 3500 significant features compared to wild-type, see strain-specific gene cards), so are well suited for high-throughput drug screens. However, *imb-2[D157N]* exhibits a moderate behavioural phenotype (770 significant features). This was somewhat surprising because the orthologous mutation on which this strain was modelled is associated with a severe ultra-rare disease in humans. Furthermore, the double deletion of *imb-2* and it’s paralogs, *imb-1* and *imb-3*, is lethal in worms [98–100]. TNPO2 variants cause a number of neuronal abnormalities. Both loss and gain of transportin activity cause developmental defects [101] and disease severity has been found to depend on the position of mutations within the protein [24, 101].

Although we detect 770 behavioural differences between *imb-2*(*syb6372*) and N2 (strain specific gene card), these are relatively small changes that are detected with a large number of well replicates (*n* > 300). Random sub-sampling of the tracking data to simulate sample sizes typical for a drug screen makes phenotype detection unreliable (Additional File 4). We therefore tested a number of screening conditions to enhance the separation of these strains in phenomic space to enable a drug screen. *imb-2*(*syb6372*) is homozygous for the aspartic acid to asparagine variant, whereas the corresponding patient variant is heterozygous. Given that heterozygous overdominance is known to impact fitness and that certain diseases only manifest in heterozygous patients, *e.g.,* PCHDH19-related epilepsy [102], we first determined the behaviour of a heterozygous variant (*imb-2[D157N]/* +). Phenotypic comparison of *imb-2[D157N]/* + and N2/ + revealed no behavioural differences between the heterozygous strains (data not shown), similar to prior findings that *imb-2*(*vy10*) (containing a different single amino acid change) is recessive in *C. elegans* [24].

**Fig. 5:**
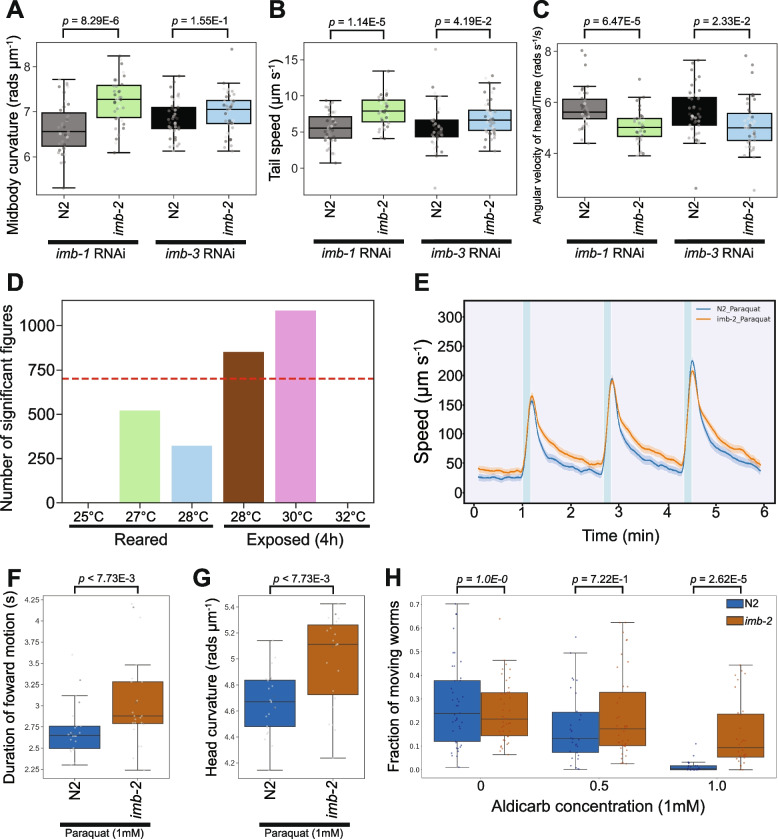
Enhancing the phenomic separation of *TNPO2* mutants and controls. **A**-**C** Key behavioural differences of *imb-2*(*syb6372*) and wild-type worms following the RNAi-mediated silencing of *imb-1* (left pair of boxes) or *imb-3* (right pair of boxes). **D** Total number of behavioural differences detected between *imb-2*(*syb6272*) and N2 reared from the L1 stage at higher temperatures (25 °C, 27 °C or 28 °C, left 3 bars) or exposed to higher temperatures (28 °C, 30 °C or 32 °C) for 4 h prior to tracking (right 3 bars). Red dashed line denotes the number of phenotypic changes detected between *imb-2*(*syb6372*) and wild-type reared at 20 °C without any treatment. Speed of worms upon stimulation with three 10 s pulses of high intensity blue light (blue shaded regions), each 100 s apart. Both strains were treated with 1 mM paraquat for 4 h prior to tracking. Coloured lines represent average speed of the detected worms across all biological replicates and shaded areas represent 95% confidence intervals. **F**-**G** Key behavioural differences of strains treated with 1 mM paraquat (as described in 5E). **H** Fraction of worms moving after 4 h exposure to 0.5 or 1 mM aldicarb. Individual points marked on the box plots are well averaged values (3 – 5 worms per well) for each feature across the independent days of tracking. *p*-values are for comparison to wild-type N2, treated with the same stressor condition, using block permutation t-tests (*n* = 10,000 permutations, correcting for multiple comparisons using the Benjamini- Yekutieli method). For all conditions, data represents *n* > 23 wells

Alongside *imb-2*, *C. elegans* encodes *imb-1* (*KPNB1* ortholog) and *imb-3* (*IPO5* and *RANBP6* ortholog) that also play a role in the import of proteins into the nucleus [103–106]. Due to potential functional redundancy, we used RNA interference (RNAi) to knock down *imb-1* or *imb-3* in *imb-2* mutants (Fig. 5A-C). We fed *imb-2* mutants bacteria expressing dsRNA targeting *imb-1* and *imb-3* as larvae and tracked worms as adults. There was not a large effect of RNAi, but focusing on the subset of features highlighted in the *imb-2* gene card (Additional File 1), silencing of *imb-1* resulted in the best separation of strains with respect to increased midbody curvature, increased tail speed, and decreased head foraging (time derivative of angular velocity) behaviours (Fig. 5A-C). Still, sub-sampling the tracking data to simulate a drug screen with *n* = 3 replicates shows that the phenotype is not robust (Additional File 5).

Next, we used temperature as a candidate sensitiser. Rearing both strains from the L1 stage at higher temperatures (25 °C, 27 °C or 28 °C, Fig. 5D) leads to a reduction in the number of behavioural differences between the strains. Exposing adult worms to 28 °C for 4 h prior to tracking resulted in negligible difference in the separation of mutant and wild-type strains. 4 h exposure of *imb-2[D157N]* and N2 to 32 °C causes all worms to become near-stationary and there is little detectable difference. Although a comparison of worms exposed to 30 °C for 4 h enhances the *imb-2*(*syb6372*) phenotype, this may simply reflect a greater variability in these conditions since most of the differences relate to feature interquartile ranges rather than their medians.

Following temperature, we tested how chemical perturbation with paraquat or aldicarb affected behaviour. Paraquat is a herbicide known to induce oxidative stress and behavioural changes in *C. elegans* [107, 108]. We find that exposure of mutant and wild-type strains to paraquat (1 mM for 4 h) results in 1907 significant behavioural differences between *imb-2*(*syb6372*) and N2. The majority of these new behavioural differences related to speed-associated features, with the mutant exhibiting a higher pre-stimulation (baseline) speed and a more sustained increase in speed following stimulation with blue light (Fig. 5E). Furthermore, we see an increase in the overall duration of movement upon the blue light stimulation of *imb-2*(*syb6372*) (Fig. 5F) and a greater separation of curvature related features (Fig. 5G).

Treatment with aldicarb causes an accumulation of acetylcholine in the synaptic cleft of the neuromuscular junction, leading to eventual paralysis, and is commonly used to identify altered synaptic transmission in *C. elegans* mutants [109]. There is no difference in the baseline movement of solvent treated worms, yet there is a dose-dependent decrease in the fraction of worms moving (increased paralysis) upon aldicarb treatment (Fig. 5H). Treatment with 1 mM aldicarb for 4 h causes near-complete paralysis of N2 while *imb-2[D157N]* mutants are more likely to move, showing some aldicarb resistance. This suggests decreased levels of synaptic transmission for *imb-2[D157N]* variants as acetylcholine accumulates more slowly in the synaptic cleft and that aldicarb may be a useful sensitiser for phenotypic screens.

### Mutation in cohesin subunit SMC-3

Cornelia de Lange syndrome (CdLS) is a genetic syndrome characterised by variable neurodevelopmental defects, facial dysmorphism, upper limb anomalies and atypical growth [110]. Most CdLS patients carry heterozygous mutations in different subunits of the cohesin complex, a molecular motor that is involved in chromosome segregation, DNA repair, and 3D genome organization [111]. How mutations in cohesin subunits cause CdLS is not fully understood, but it is thought that reduced cohesin function caused by heterozygous mutations induces defects in 3D genome organisation that alter the expression of developmental genes [112]. In contrast, homozygous mutations that fully eliminate cohesin function are embryonically lethal, likely due to the essential role of cohesin in ensuring accurate chromosome segregation during cell division.

Genetic testing in individuals with clinical features that may be consistent with CdLS may identify de novo non-synonymous variants in cohesin subunits, including SMC3 [113]. However, many identified variants are currently classified to be of uncertain clinical significance (VUS), for example if they have not been previously reported in other affected individuals. Often, the information that is currently available about the functional impact of a specific variant is limited.

**Fig. 6:**
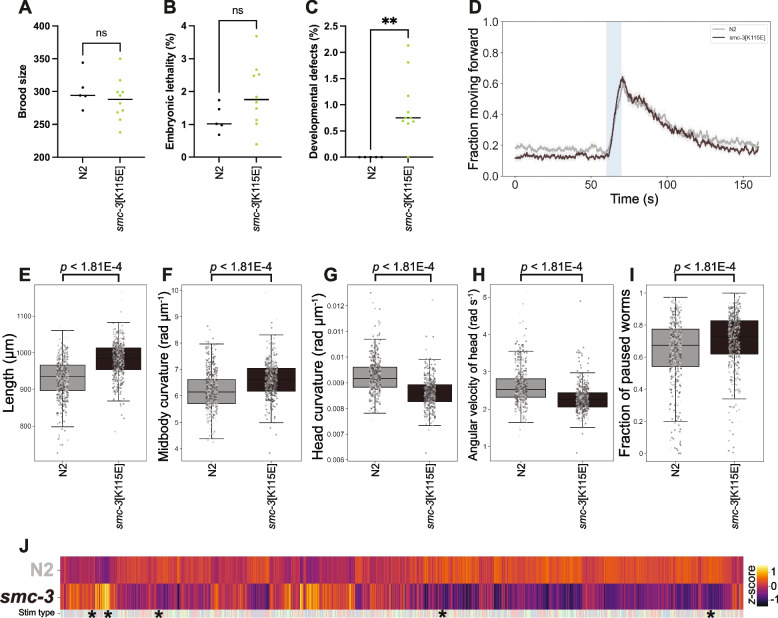
Patient-specific *smc-3[K115E]* mutants display behavioural defects. Quantification of brood size laid by wild-type controls (*n* = 5) and *smc-3[K115E]* mutant worms (*n* = 10). *p* = 0.49 by a two-tailed Mann Whitney test. **B** Quantification of embryonic lethality amongst the broods displayed in (**A**). *p* = 0.10 by a two-tailed Mann Whitney test. **C** Quantification of the frequency of developmental defects amongst the broods displayed in (A). *p* = 0.0033 by a two-tailed Mann Whitney test. **D** Overall fraction of worms moving forward 60 s prior to and 80 s following stimulation with a 10 s blue light pulse (blue shading). Coloured lines represent averages of the detected fraction of paused worms across all biological replicates (*n* > 500) and shaded areas represent 95% confidence intervals. **E**-**I** Key behavioural features altered between N2 and the *smc-3 K11E* mutant. Individual points marked on the box plots are well averaged values (3 – 5 worms per well) for each feature across the independent days of tracking (*n* > 500). *p*-values are for comparison to wild-type N2 using block permutation t-tests (*n* = 100,000 permutations, correcting for multiple comparisons using the Benjamini-Yekutieli method). **J** Heatmap of the entire set of 8289 behavioural features extracted by Tierpsy. The ‘stim type’ barcode denotes when during image acquisition a feature was extracted: pre-stimulation (pink), blue light stimulation (blue) and post-stimulation (green). Asterisks show the location of the selected features presented in E-I

To help explore this issue, we tested whether an SMC3[K114E] mutation identified as a VUS in an individual with a clinical presentation compatible with CdLS would result in any phenotypic defects in worms. The K114 residue is close to the ATPase head domain of SMC3 and corresponds to K115 in *C. elegans* SMC-3, thus we used CRISPR to create a *C. elegans* strain carrying the SMC-3[K115E] mutation. Similar to mammals, complete loss of cohesin function is lethal in *C. elegans* due to impaired chromosome segregation during embryonic development [45]. We first set up to determine whether the SMC-3 K115E mutations may represent a LoF mutation by investigating the brood size and viability of homozygous *smc-3[K115E]* mutants. Quantification of these two parameters revealed no significant differences between homozygous *smc-3[K115E]* mutant worms and wild-type controls (Fig. 6A-B), suggesting that the SMC-3[K115E] mutation does not induce defects in chromosome segregation that compromise embryonic viability.

Despite this, we observed that a small, but significant, percentage of progeny from homozygous *smc-3[K115]* homozygous mutants displayed different developmental defects including: larval arrest, vulva protrusion, and abnormal body shape (Fig. 6C). This suggested that the SMC-3[K115E] substitution does impact normal development, prompting us to investigate its potential effect on behavioural phenotypes. Tracking of adult homozygous *smc-3[K115]* mutant worms revealed strong behavioural phenotypes with 5685 significant behavioural differences compared to wild-type controls (Fig. 6D-J). *smc-3[K115E]* worms are longer and have increased midbody curvature, but decreased curvature and angular velocity of the head. The patient avatar is less active during baseline recordings but has a normal response to stimulation with blue light. These results demonstrate that the SMC-3[K115E] substitution in *C. elegans* contributes towards developmental and behavioural defects without impacting on embryonic viability, mimicking the phenotype observed in CdLS patients and suggesting that the K114E in human SMC3 may contribute to the CdLS-like phenotypes observed in the heterozygous patient.

## Discussion

There are more than 3000 conserved *C. elegans* genes that currently have an association with human disease [7]. This number will grow as more patients are sequenced and the number of potential disease models is greater still since there may be multiple variants per gene that warrant separate modelling efforts. The high-throughput phenotyping of worm disease models provides an opportunity to map this extensive genotype–phenotype space.

For the majority (22/25) of the mutants generated in this study, we focused on deletions of large coding regions to give high-confidence LoF mutations. This approach was chosen to quickly assess if these often-understudied genes might be suitable for the creation of patient-specific disease model variants in the future. It is important to acknowledge that this approach is not without limitations and, in some cases, complete deletion of a genetic target may complicate the interpretation of phenotyping. Complete deletion of a gene may trigger compensatory mechanisms when compared to partial deletion or the introduction of early stop codons. As an example, *mec-10*(*tm1552*) mutants (deletion mutant) can sense touch due to compensation by *mec-4* [114]. Whereas *mec-10*(*e1515*) and *mec-10*(*u20*) mutants (other LoF alleles) are touch insensitive. Similarly, modelling patient specific *AGO1* mutations in *alg-1* (worm ortholog) results in stronger behavioural phenotypes compared to *alg-1* null mutants [115]. At the same time, we found clear phenotypes for *pde-1* and *pde-5* LoF mutants (strain-specific gene cards) despite previous findings that PDE-1 and PDE-5 are partially redundant and single mutants show only mild thermotaxis defects [116]. Although not a perfect model for all pathological mutations, full deletions can provide a first-pass strategy for identifying candidate genes to warrant future functional analysis.

Although we detected differences in all 25 of the strains in this study, three mutants (*imb-2*(*syb6372*), *shl-1*(*syb5907*), and *tmem-222*(*syb4882*)) showed weak to moderate phenotypes (< 1000 significant feature differences) compared to wild-type animals. Strains with weak phenotypes are challenging to use in drug repurposing screens where the number of replicates per treatment is likely to be small. In previous work, a chemical sensitiser caused an easily measurable phenotype that was used in a successful drug repurposing screen [117].

We treated *imb-2(syb6372*) mutants with different temperatures, RNAi against related genes, and two compounds with different modes of action. We chose these methods because they are compatible with high-throughput screening. Other candidates for sensitisation include optogenetic stimulation of specific neural circuits and mechanical stimuli delivered to the entire plate. Other kinds of behavioural assays such as chemotaxis that might draw out novel phenotypes are more difficult to perform with high-throughput, although it is an area of active research [118]. RNAi resistance is a previously reported *imb-2* phenotype [119], so it is perhaps unsurprising that we were unsuccessful in using targeted genetic sensitisation methods to enhance our *imb-2[D157N]* mutant phenotype. Although the site of the mutated amino acid is conserved between humans and *C. elegans*, BLAST analysis reveals only 52.67% identity similarity between these orthologs. Hence, insufficient conservation of the gene product could explain the lack of a clearly observed phenotype seen for this *C. elegans* patient avatar. Nonetheless, we remain optimistic that the specificity of RNAi and the ease of delivery through feeding will be useful in other cases to sensitise disease models.

Likewise, chemical perturbation is easily incorporated into this phenotyping approach and may help to identify additional cellular pathways that are affected by disease mutations. For example, 4 h aldicarb treatment led to a clear separation between *imb-2*(*syb6372*) and N2. This could enable future drug screens to be performed by identifying compounds that rescue the paralysis phenotype, hence pushing the behaviour of the mutant towards the healthy control. A caveat of this approach is that drugs that themselves caused paralysis or had broad toxic effects would show up as hits and need to be filtered with a counter screen. At the same time, aldicarb resistance in *imb-2[D157N]* worms suggests a slower accumulation of acetylcholine in the synaptic cleft [109], a phenotype that would not have been obvious from tracking in unperturbed conditions. Similarly, we identify that FLCN deficiency may lead to an increased susceptibility of mTORC2 inhibition, whereas FNIP1/2 deficiency may display a greater susceptibility to MTOR1/2 inhibition.

Overall, our results further support the use of high-throughput tracking to phenotype diverse worm disease models, extending our previous results on knockouts to more models, including patient-specific single amino acid changes and heterozygous mutants. Similar to the knockout strains, several of the point mutants showed strong phenotypes that could support drug repurposing screens. The relative ease and low cost of generating new worm models, coupled with the simplicity and ease of phenotyping them, means a systematic search for treatments to hundreds of Mendelian diseases is within reach.

## Conclusion

Our study demonstrates the power of high-throughput behavioural phenotyping in *C. elegans* to systematically characterise diverse disease-associated gene variants, including patient-specific mutations. By extending previous knockout-based analyses to include loss-of-function and missense variants, we establish a scalable platform for evaluating the phenotypic consequences of human disease alleles. While complete gene deletions have limitations, they offer a rapid, first-pass approach to identify promising candidate genes for deeper mechanistic or therapeutic investigation. Importantly, we show that phenotypic sensitisation through chemical or genetic perturbation can uncover otherwise cryptic phenotypes and expand the utility of weak models for future compound screening. Together, our findings reinforce the utility of *C. elegans* as a versatile and cost-effective model for disease research and lay the groundwork for future large-scale efforts to functionally annotate variants of uncertain significance and support therapeutic discovery across a broad spectrum of genetic disorders.

## Methods

### Mutant generation

To generate loss-of-function mutants, CRISPR guide RNAs were designed to target large deletions that start close to the start codon and excise several exons from a gene to yield high confidence loss of function of the entire gene. For patient-specific allelic variants, guide RNAs were designed to target a single *C. elegans* codon corresponding to the position of the desired amino acid change in humans. Mutants were designed, made and confirmed by sequencing in N2 background by SunyBiotech. The strains made by SunyBiotech were not outcrossed. Transgenic worms carrying the patient variant SMC3 Lys114Glu were generated by in vitro assembly of Cas9 ribonucleoprotein complexes and a ssDNA repair template containing the desired amino acid change franked with short sequences (35 bp) homologous to the target region [120]. The ATATTACATTGATAATAAAA sgRNA was used to target the desired *smc-3* region and the strain was outcrossed 3 times. A list of all mutant strains used in this study can be found in Table 1.

**Table 1:** List of *C. elegans* strains used in this study

Gene	Strain Name	Wild-type Gene Length	Mutation
*blos-1* *(syb6895)*	PHX6895	954 bp	Deletion of entire coding region: 927 bp deletion starting at nucleotide (nt) 9
*blos-8* *(syb6686)*	PHX6686	1280 bp	Deletion of entire coding region: 1790 bp deletion starting at nt 10
*blos-9* *(syb7029)*	PHX7029	PHX7029 1046 bp	Deletion of first half of exon 1 and remaining coding regions: 945 bp deletion starting at nt 49
*ccpp-1* *(syb4843)*	PHX4843	6201 bp	Deletion of entire coding region: 6180 bp deletion starting at nt 21
*cpx-1* *(syb7694)*	PHX7694	1568 bp	Deletion of entire coding region: 1196 bp deletion starting at nt 41
*flcn-1* *(syb8071)*	PHX8071	18087 bp	Deletion of exon 1 to exon 8: 17582 bp deletion starting at nt 296
*fnip-2* *(syb8038)*	PHX8038	13091 bp	Deletion of entire coding region: 23685 bp deletion starting at nt 135
*imb-2* *(syb6372)*	PHX6372	931 bp	Single amino acid polymorphism: D to N substitution at amino acid position 157
*irk-1* *(syb5903)*	PHX5903	11931 bp	Deletion of entire coding region: 11338 bp deletion starting at nt 168
*let-526* *(syb8759)*	PHX8759	1003 bp	Heterozygous deletion of entire coding region, balanced using hT2(I, III): 9024 bp deletion starting at nt 91
*ncap-1* *(syb4936*	PHX4936	4624 bp	Deletion from first half of exon 1: 4252 bp deletion starting at nt 166
*odr-8* *(syb4940)*	PHX4940	3171 bp	Deletion from first half of exon 1: 2585 bp deletion starting at nt 39
*pacs-1* *(syb7634)*	PHX7634	1166 bp	Single amino acid polymorphism: E to K substitution at amino acid position 205
*pde-1* *(syb7700)*	PHX7700	7830 bp	Deletion of entire coding region: 7095 bp deletion starting at nt 40
*pde-5* *(syb7506)*	PHX7506	4025 bp	Deletion of entire gene: 4025 bp deletion starting at nt 1
*pmp-4* *(syb7777)*	PHX7777	3047 bp	Deletion of entire gene: 3047 bp deletion starting at nt 1
*R10E11.6* *(syb7507)*	PHX7507	2858 bp	Deletion of entire coding region: 2488 bp deletion starting at nt 26
*rpy-1* *(syb5027)*	PHX5027	3603 bp	Deletion of entire coding region: 3054 bp deletion starting at nt 26
*sam-4* *(syb6765)*	PHX6765	1305 bp	Deletion from first half of exon 1: 757 bp deletion starting at nt 234
*sec-31* *(syb6825)*	PHX6825	5815 bp	Deletion of entire coding region: 5775 bp deletion starting at nt 40
*shl-1* *(syb5907)*	PHX5907	5589 bp	Deletion of exon 1 to exon 5: 5166 bp deletion starting at nt 91
*smc-3* *(fq180)*	*fq180[K115E]* *III*	4003 bp	Single amino acid polymorphism: K to E substitution at amino acid 115
*tmem-222* *(syb4882)*	PHX4882	657 bp	Deletion of entire coding region: 544 bp deletion starting at nt 16
*vps-50* *(syb6653)*	PHX6653	5137 bp	Deletion of entire coding region: 5111 bp deletion starting at nt 201
*Y47D9A.1* *(syb7612)*	PHX7612	983 bp	Single amino acid polymorphism: R to W substitution at amino acid 298

### C. elegans preparation

For standard phenotyping experiments, all strains were cultured on Nematode Growth Medium (NGM) at 20 °C and fed with *E. coli* OP50 following standard procedure [121]. Synchronised populations of young adult worms were used for imaging and were cultured by bleaching unsynchronised gravid adults and allowing L1 diapause progeny to develop for 2.5 days at 20 °C (detailed protocol: 10.17504/protocols.io.2bzgap6). *let-526*(*syb8759*) and *odr-8*(*syb4940*) were developmentally delayed and allowed to grow for longer (6 h and 7 h, respectively) before imaging. Before imaging, young adults were washed in M9 (detailed protocol: 10.17504/protocols.io.bfqbjmsn) and transferred to tracking plates (3–5 worms per well) using a COPAS 500 Flow Pilot (detailed protocol: 10.17504/protocols.io.bfc9jiz6), before being returned to a 20 °C incubator for 3.5 h. Plates were transferred onto the multi-camera tracker for another 30 min to habituate prior to imaging (detailed protocol: 10.17504/protocols.io.bsicncaw).

### Compound screening

For the chemical treatment of *imb-2*(*syb6372*), aldicarb and paraquat were dissolved in ddH_2_O. The day prior to tracking, imaging plates were dosed to achieve the desired final well concentration of each compound (1 mM paraquat and 0.5 or 1 mM aldicarb) prior to seeding with bacteria (see below for details). Plates were left to dry (~ 30 min) and then stored in the dark at room temperature overnight. Using the methods above, *imb-2*(*syb6372*) and N2 worms (age synchronized young adults) were dispensed into the imaging plate wells and incubated at 20 °C for 4 h before tracking.

*E. coli* OP50 treated with 0.5% paraformaldehyde for 2 h [94], then washed 3 times in M9 buffer, was used as the food source for the folliculin mutant drug screens (detailed protocol:10.17504/protocols.io.81wgbyq71vpk/v1). All compounds acting on the AMPK/mTOR pathways were dissolved in DMSO. The day prior to tracking, imaging plates were dosed with the compounds to achieve a final well concentration of 100 µM prior to seeding with PFA-treated *E. coli* OP50. N2, *flcn-1*(*syb8071*) and *fnip-2*(*syb8038*) were all exposed to the compounds, or an identical volume of DMSO only (1% w/v), for 4 h prior to tracking (as described above).

### Increased temperature screening

When comparing the behaviour of *imb-2*(*syb6372*) and N2 at increased temperatures, worm populations were age synchronised as described above. For rearing the strains at higher temperatures, L1 worms were refed onto plates seeded with *E. coli* OP50 and incubated at 25 °C, 27 °C or 28 °C. Worms were then washed and dispensed into tracking plates and incubated for 3.5 h at their rearing temperature. 30 min prior to imaging, plates were transferred to the multi-camera tracker and allowed to habituate. For the acute exposure of strains to increased temperatures, *imb-2*(*syb6372*) and N2 were reared at 20 °C. On the day of tracking, young adults were dispensed into tracking plates that were incubated at 28 °C, 30 °C and 32 °C for 4 h before being immediately imaged.

### RNAi silencing of imb-1 and imb-3

*E. coli* HT115(DE3) expressing *imb-1* or *imb-3* target-gene dsRNA from the Ahringer RNAi Feeding Library [122] were used as a food source on tracking and nursery plates used to rear age synchronised *imb-2*(*syb6372*) and N2 worms to adulthood. Both NGM and no-peptone NGM was supplemented with tetracycline (15 µg mL^−1^, Sigma) and ampicillin (100 µg mL^−1^, Sigma) for microbial selection, alongside 1 mM isopropylthio-β-galactoside (IPTG, Thermo Fisher) to induce dsRNA production. Bacterial cultures were grown overnight (37 °C, 200 rpm shaking) in LB (Miller) supplemented with tetracycline and ampicillin. Cultures were used to inoculate fresh LB (OD_600_ 1.0) supplemented with 1 mM IPTG and incubated at 37 °C until OD_600_ 1.0 was achieved (~ 2 h). Density-normalised cultures were cooled to 20 °C and used in place of *E. coli* OP50, all other methods remain the same.

### Imaging plate preparation

No-peptone NGM was used for worm tracking plates (detailed protocol: 10.17504/protocols.io.bvian4ae). Briefly, 20 g agar (Difco) and 3 g NaCl was dissolved in 975 mL ddH_2_O and autoclaved. Once molten agar was cooled to 50–60 °C post-autoclave salts (1 mL CaCl_2_ [1 M]; 1 mL MgSO_4_ [1 M]; 25 mL KPO_4_ [1 M, pH 6.0]) and cholesterol (1 mL of 5 mg mL^−1^ stock) were added. Then 200 mL of agar was dispensed into each well of 96-square well plates (Whatman UNIPLATE: WHAT-77011651) using an Integra VIAFILL (detailed protocol: 10.17504/protocols.io.bmxbk7in). Poured plates were stored in an air-tight container, agar side up at 4 °C until required. One day before imaging, plates were dried in a cabinet to lose 3–5% of their weight by volume. An Integra VIAFILL dispenser was then used to seed the wells of each plate with 5 mL of bacterial food (OD_600_ 1.0).

### Image acquisition, processing and feature extraction

All videos were recorded and features extracted using methods we have previously described in detail [7]. Briefly, videos were acquired at 25 frames per second in a temperature-controlled room (20 °C) using a shutter time of 25 ms and a resolution of 12.4 µm/pixel. Three sequential videos were recorded: a 5-min pre-stimulus video; a 6-min blue light recorded with three 10-s pulses of high intensity blue light 100-s apart (starting after 60 s); and a 5-min post-stimulus recording. Videos were segmented and tracked using Tierpsy Tracker [9] and a convolutional neural network classifier was used filter out non-worm objects [8]. We further filtered the data to only keep worm skeletons with a length of 700–1300 mm and width of 20–200 mm before using Tierpsy Tracker’s viewer to exclude wells with visible contamination, agar damage, compound precipitation, or excess liquid from downstream analysis. A previously-defined set of 3076 behavioural features [10] were extracted for each well in the three videos (pre-stimulus, blue light and post-stimulus) and the feature values were averaged to give a single feature vector for each well (*n* = 1).

### Statistical analysis

Significant differences in the behavioural feature sets extracted from each model strain were compared to the N2 reference, for each of the 3 video recordings, using block permutation t-tests (https://github.com/Tierpsy/tierpsy-tools521/python/blob/master/tierpsytools/analysis/statistical_tests.py). Python (version 3.9.2) was used to perform the analysis, using *n* = 100,000 permutations that were randomly shuffled within the independent days of image acquisition (to account for day-to-day variation across day replicates). The resulting *p*-values were corrected for multiple comparisons using the Benjamini-Yekutieli method, with a false discovery rate of 5% [123]. The same methods were used to compare differences in the behaviour of *imb-2*(*syb6372*) and N2 treated with the same compounds, RNAi or the same change in temperature. For the folliculin mutant drug screens, statistical differences were calculated using the Kruskal–Wallis test and correcting for multiple comparisons using the Benjamini-Yekutieli method, between the DMSO-only (untreated) and compound-treated strains. Heatmaps, cluster maps and principal component analysis of the extracted feature sets for each strain compared to the N2 reference were calculated using Seaborn (0.11.1) packages [124]. All scripts used for statistical analysis and the generation of figures are available in a project specific repository, available here: 10.5281/zenodo.16420562.

## Supplementary Information

Additional file 1. Strain-specific cards. Detailing: phenotype summaries; the molecular characteristics; previously reported phenotypes (if available) and human disease(s) associated with each strain studied.

Additional file 2. Principal component analysis of AMPK and mTOR inhibition or activation on folliculin mutant phenotypes. PCA of the folliculin disease model mutants and wild-type strains treated with DMSO only (stars) and the same strains treated with 100 µM of each compound (different shaped, darker makers) for 4 h prior to tracking (*n* > 17). Variance explained by the components is denoted in brackets and error bars represent standard deviation. (A) First 2 principal components of the entire behavioural feature set extracted by Tierpsy, (B) third and further principal components. PCA analysis reveals that treatment with the AMPK or mTOR inhibitor/activators tested did not restore the mutant phenotypes to wild-type. However, treatment with the different compounds moves the wild-type and mutant strains in the same direction within phenotypic space.

Additional file 3. Western blots of mTOR and AMPK activity. Changes in AMPK and mTOR signalling were monitored using antibodies against P-T172 AMPK and P-S49 eIF2α respectively using protein lysates of N2, *flcn-1* and *fnip-2* worms treated with the indicated drugs at the same concentrations used for behavioural study. Blots show technical replicates representative of (*n* = 5), each condition was monitored with two blots, one shown here. Western blotting was performed as described in additional methods (Additional File 6). β Actin was used as loading control.

Additional file 4. TNPO2 patient avatar phenotypes cannot be reliably detected with a low number of well replicates. (A-E) Sub-sampled boxplots of the same behavioural feature (midbody curvature) previously detected as being statistically significant between *imb-2*(*syb6372*) and wild-type when using a large number of replicates (*n* > 300, strain specific gene card). Individual plots show randomly sampled data points (*n* = 3) from the same complete dataset. *p*-values are for comparison of *imb-2*[D157N] mutant to N2 using Student’s t-test, correcting for multiple comparisons using the Benjamini- Yekutieli method. (F–H) Histograms of 3 key behavioural features (shown in the strain specific gene card) showing the calculated *p*-value of each feature when sub-sampling the overall dataset to achieve *n* = 3 replicates (using same methods as for the individual boxplots). Red line shows *p* = 0.05, and is considered statistically significant. When looking at a small number of samples (mimicking what may be typically collected for a low replicate screen across a large number of compounds), the behavioural phenotype of the disease model mutant cannot be reliably distinguished from wild-type.

Additional file 5. Targeted sensitisation of TNPO2 patient avatar does not result in a reliable phenotype when using a low number of well replicates. (A-I) Key behavioural phenotype of *imb-2*(*syb6372*) and wild-type worms following the RNAi-mediated silencing of *imb-1* (left pair of boxes) or *imb-3* (right pair of boxes). Individual plots show the same feature (repeated 3 times per row) for randomly sampled points (*n* = 3) from the complete dataset (shown in Fig. 5). *p*-values are for comparison of *imb-2*[D157N] mutant to N2 using Student’s t-test, correcting for multiple comparisons using the Benjamini-Yekutieli method. As in Additional File 4, when looking at a small number of samples the behavioural phenotype of the mutant cannot be reliably distinguished from wild-type.

Additional file 6.

## Data Availability

All data generated or analysed during this study are included in this published article, its supplementary information files and publicly available repositories:https://zenodo.org/records/13941711 (datasets and associated metadata) and 10.5281/zenodo.16420562 (code).

## Abbreviations

AMPK: 5′-AMP activated protein kinase
BHD: Birt-Hogg-Dubé syndrome
BLAST: Basic Local Alignment Search Tool
BORC: BLOC-one-related complex
CdLS: Cornelia de Lange syndrome
ddH_2_O: Double distilled water
dsRNA: Double stranded ribonucleic acid
IPTG: Isopropylthio-β-galactoside
L1: Larval stage 1
LB: Lysogeny broth
LoF: Loss-of-function
mTOR: Mammalian target of rapamycin
mTORC: Mammalian target of rapamycin complex
NGM: Nematode growth medium
PCA: Principal component analysis
PFA: Paraformaldehyde
RNAi: Ribonucleic acid interference
VUS: Variant of Uncertain Significance
