# Supplementary material for: High-throughput behavioural phenotyping of 25 C. elegans disease models including patient-specific mutations
Source: BMC Biol. 2025 Sep 26;23:281. doi: 10.1186/s12915-025-02368-8 (PMC12465487; doi:10.1186/s12915-025-02368-8)

blos-1(syb6895)

Strain name: PHX6895
Wild-type gene length: 945bp
Mutation: 927bp deletion starting at nt position 9 (deletion of entire gene coding region)

C. elegans Description

Function: Involved in endosomal transport and lysosomal biogeniesis[18]; predicted to be part of the BLOC-1 complex

Expression: Excretory cell; intestine and nervous system

Previously reported phenotypes: None

Human Orthologs:

BLOC1S1

Associated disease(s):
Hermansky-Pudlak syndrome

Results

3927/8289 significant features vs N2 ($p < 0.05$, block permutation t-test, 10000 permutations)

Key phenotype(s): Shorter; decreased angular velocity, curvature, speed and acceleration of head; increased forward, but attenuated backward, photophobic escape response

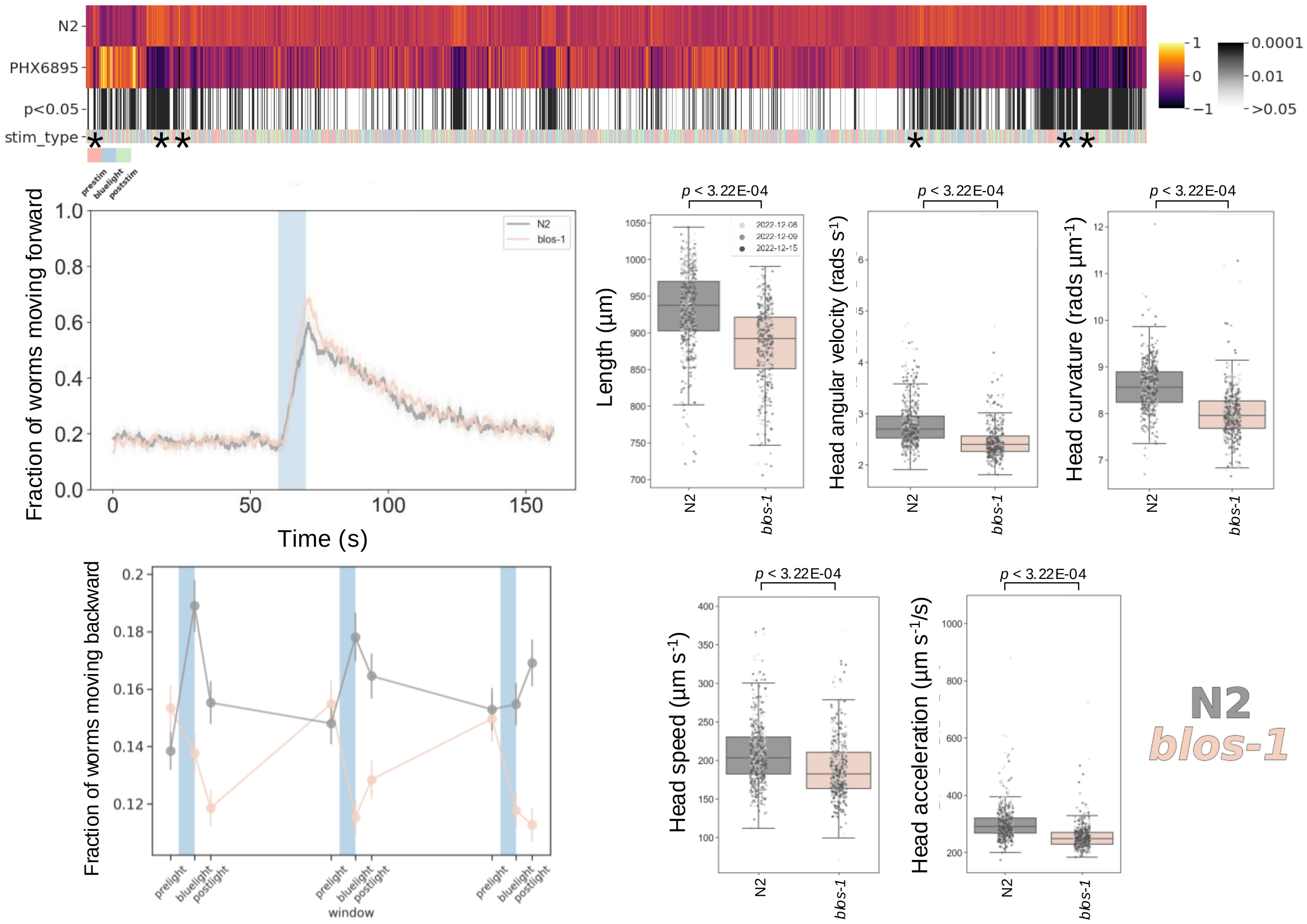

blos-8(syb6686)

Strain name: PHX6686
Wild-type gene length: 1280bp
Mutation: 179bp deletion starting at nt position 10 (deletion of entire gene coding region)

C. elegans Description

Function: Predicted to be a member of the BORC complex; associated with the cytosolic face of lysosomes[6]

Expression: Unknown

Previously reported phenotypes: None

Human Orthologs:

BORCS7

Associated disease(s):
Hermansky-Pudlak syndrome; Hereditary spastic paraplegia

Results

3034/8289 significant features vs N2 ($p < 0.05$, block permutation t-test, 10000 permutations)

Key phenotype(s): Longer; decreased angular velocity of hips; increased midbody curvature; decreased tail instantaneous angular acceleration (time derivative of angular velocity); decreased sinusoidal undulations (time derivative of midbody curvature); attenuated backward photophobic escape response

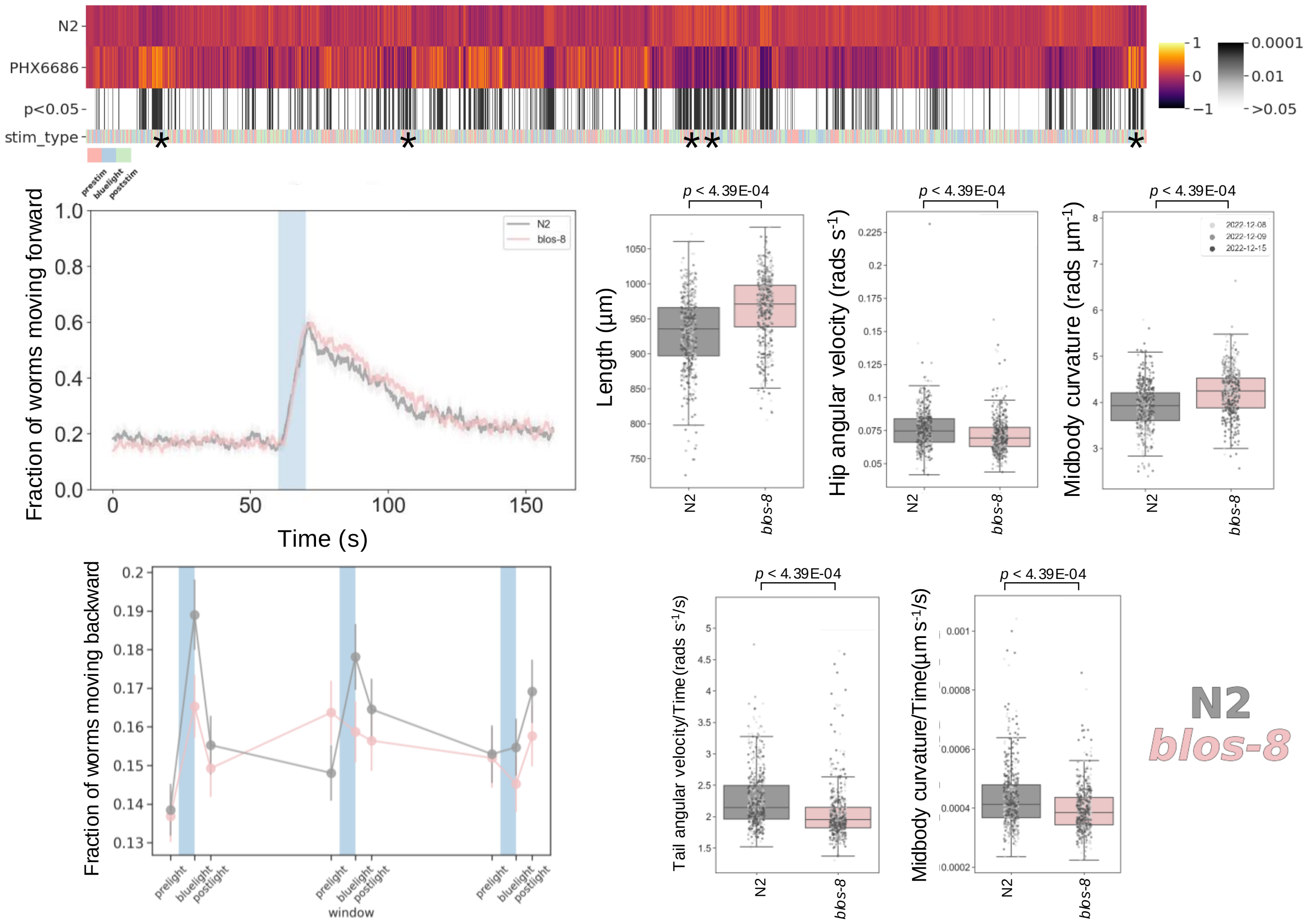

blos-9(syb7029)

Strain name: PHX7029
Wild-type gene length: 1046bp
Mutation: 945bp deletion starting at nt position 49 (half of exon 1 and remaining coding regions)

C. elegans Description

Function: Predicted to be a member of the BORC complex; associated with the cytosolic face of lysosomes[6]

Expression: Unknown

Previously reported phenotypes: None

Human Orthologs:

BORCS8

Associated disease(s):
Hermansky-Pudlak syndrome; Infantile-onset neurodegenerative disorder with altered lysosome dynamics

Results

3924/8289 significant features vs N2 ($p < 0.05$, block permutation t-test, 10000 permutations)

Key phenotype(s): Shorter; decreased angular velocity, curvature, speed and acceleration of head; attenuated forward, but increased backward, photophobic escape response

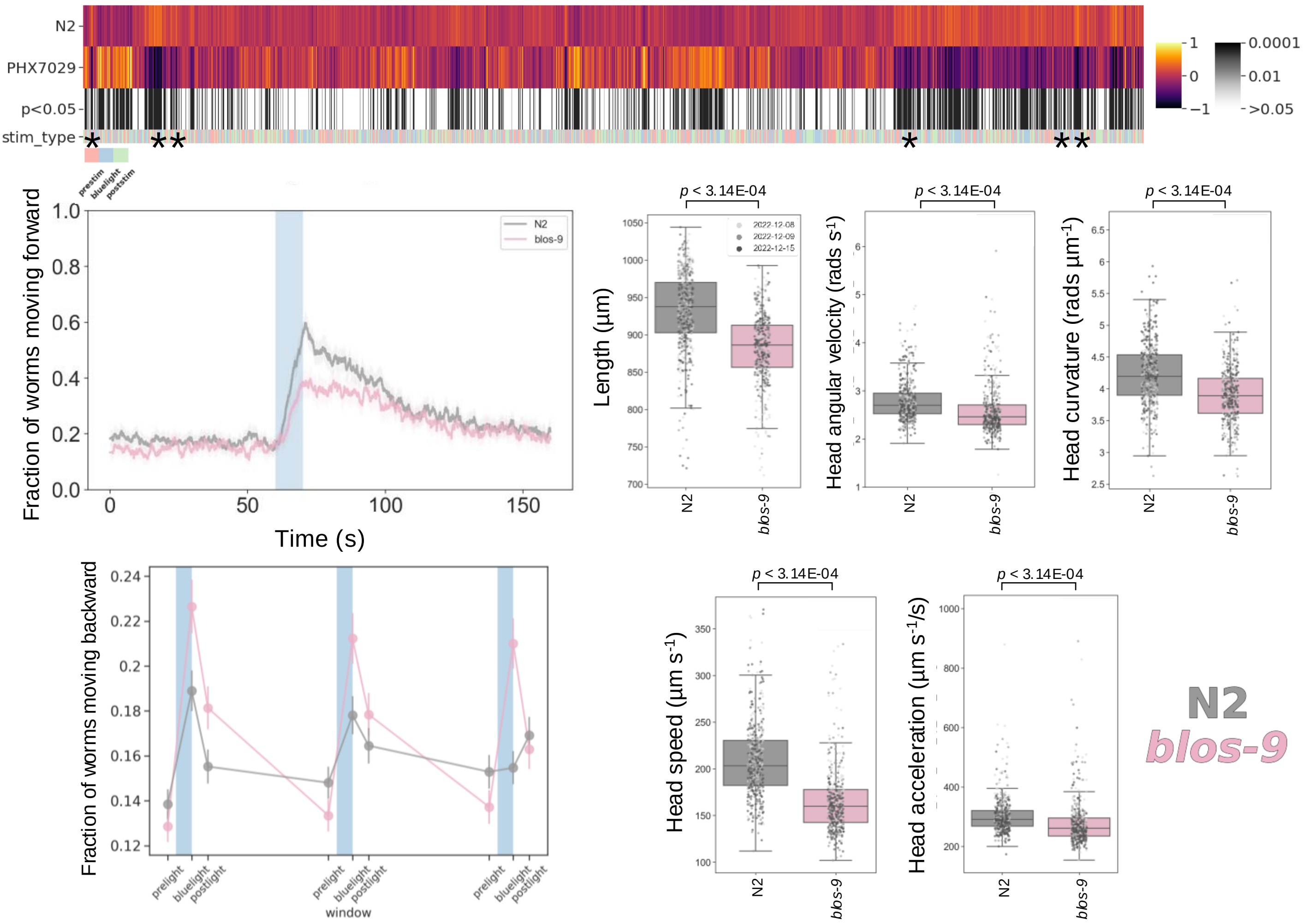

ccpp-1(syb4843)

Strain name: PHX4843
Wild-type gene length: 6201bp
Mutation: 6180bp deletion starting at nt position 21 (deletion of entire gene coding region)

C. elegans Description

Function: Predicted to enable metallocarboxypeptidase activity and zinc ion binding activity; required for ciliary maintenance but not ciliogenesis[19]

Expression: Ciliated neurons and gubernacular muscle. Predicted to be located in cilium; dendrite and perikaryon

Previously reported phenotypes: Defective ciliary localization[20]; reduction of male mating efficiency and response of males to touch[20]

Human Orthologs:

AGBL1

Associated disease(s):
Fuchs' endothelial dystrophy

Results

2592/8289 significant features vs N2 ($p < 0.05$, block permutation t-test, 10000 permutations)

Key phenotype(s): Shorter; decreased angular velocity of midbody, neck and tail while moving backwards; lower frequency midbody bends (time derivative of midbody curvature); decreased overall speed; attenuated backing in response to blue light (but no change in forward photophobic escape response)

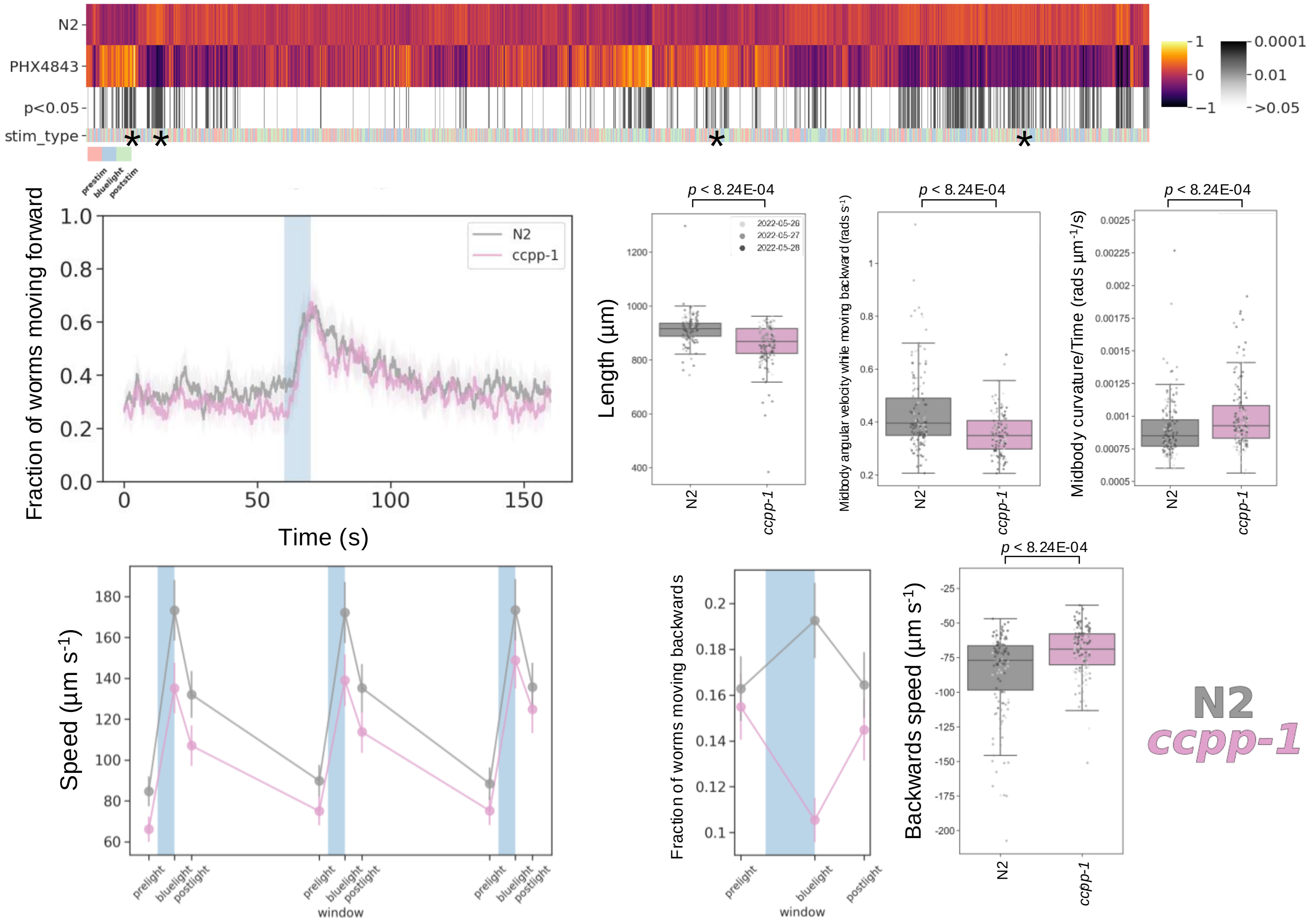

cpx-1(syb7694)

Strain name: PHX7694
Wild-type gene length: 1568bp
Mutation: 1196bp deletion starting at nt position 41 (deletion of entire gene coding region)

C. elegans Description

<u>Function</u>: Enables SNARE binding activity; involved in the regulation of neurotransmitter secretion and synaptic vesicle exocytosis

<u>Expression</u>: Head neurons; motor neurons; tail neurons; ventral cord neurons

<u>Previously reported phenotypes</u>: aldicarb hypersensitivity[21]; defective for thrashing in liquid[21]

Human Orthologs:

CPLX1 and CPLX2

<u>Associated disease(s):</u>
Developmental and epileptic encephalopathy; infantile myoclonic epilepsy; intellectual disability; Alzheimer's disease; Parkinson's disease

Results

5801/8289 significant features vs N2 (*p* < 0.05, block permutation t-test, 10000 permutations)

Key phenotype(s): Shorter; less active, with attenuated photophobic escape response; increased angular velocity and decreased curvature (for all body segments); decreased speed; increased frequency of body bends (time derivative of midbody curvature)

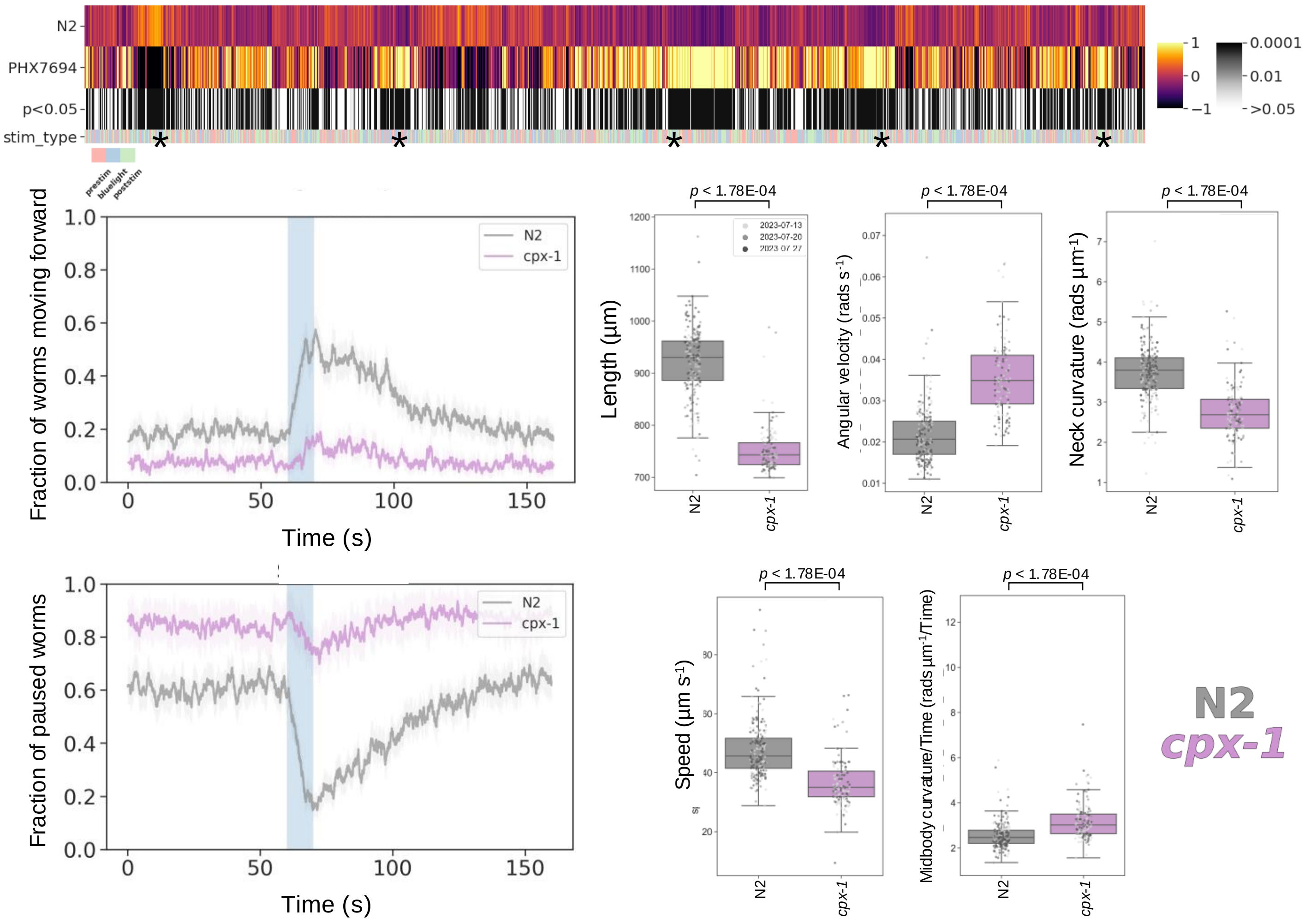

flcn-1(syb8071)

Strain name: PHX8071
Wild-type gene length: 18087bp
Mutation: 17582bp deletion starting at nt position 296 (from start of exon 1 to end of exon 8)

C. elegans Description

Function: Contributes towards GTPase activator activity; negative regulation of RNA polymerase II transcription; positive regulation of TORC1 signalling and transforming growth factor beta receptor signalling

Expression: excretory cell; nervous system; spermatheca; vulva

Previously reported phenotypes: Increased resistance to hyperosmotic stress[22]; increased glycogen content[22]; increased lifespan[23]

Human Orthologs:

FLCN

Associated disease(s):
Birt-Hogg-Dube syndrome; primary spontaneous pneumothorax; renal cell carcinoma

Results

3663/8289 significant features vs N2 ($p < 0.05$, block permutation t-test, 10000 permutations)

Key phenotype(s): Hyperactive; increased duration of forward and backward locomotion in response blue light stimulation; increased speed; increased angular velocity (all body segments); deeper and more frequent body bends (time derivative of angular velocity or curvature, respectively)

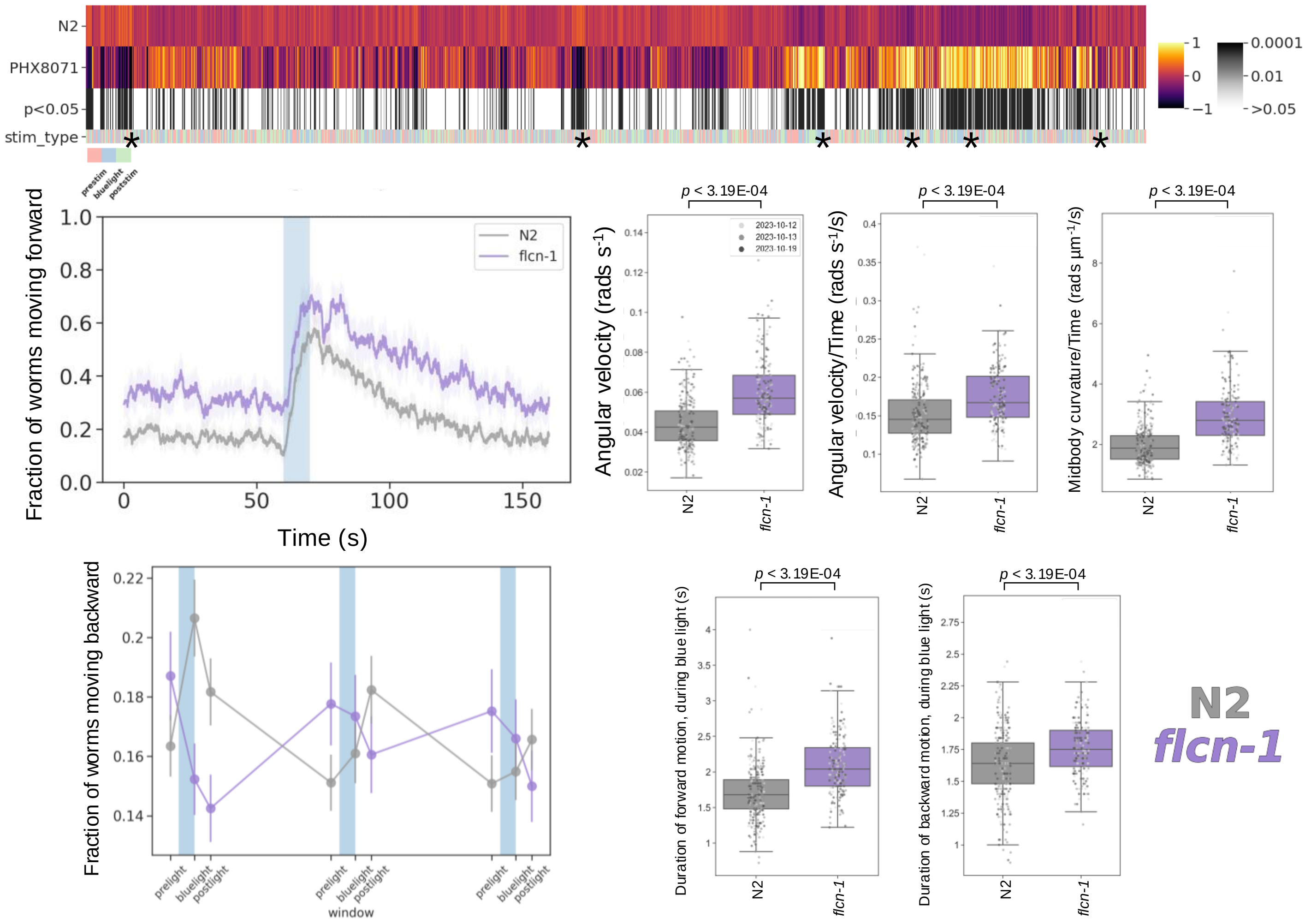

fnip-2(syb8038)

Strain name: PHX8038
Wild-type gene length: 13091bp
Mutation: 12685bp deletion starting at nt position 135 (deletion of entire gene coding region)

C. elegans Description

<u>Function</u>: Enables ATPase inhibitor activity and protein-folding chaperone binding activity

<u>Expression</u>: Predicted to be located in cytoplasm

<u>Previously reported phenotypes</u>: None

Human Orthologs:

FNIP1 and FNIP2

<u>Associated disease(s):</u>
Primary immunodeficiency disease; obesity

Results

3433/8289 significant features vs N2 (*p* < 0.05, block permutation t-test, 10000 permutations)

Key phenotype(s): Hyperactive; short; decreased duration of backward locomotion in response blue light stimulation; increased angular velocity (all body segments); increased speed; deeper and more frequent body bends (time derivative of angular velocity or curvature, respectively)

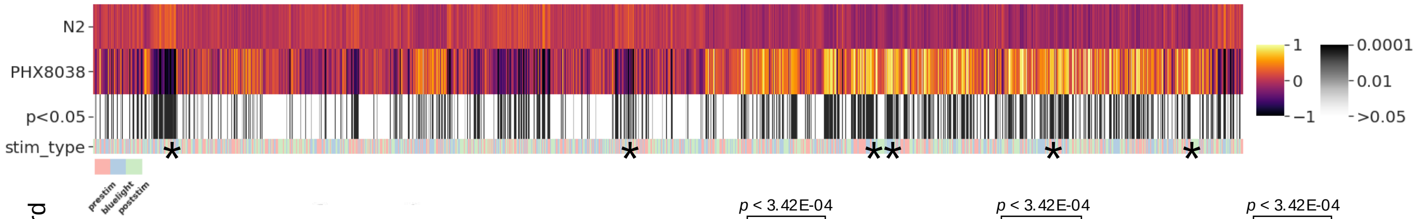

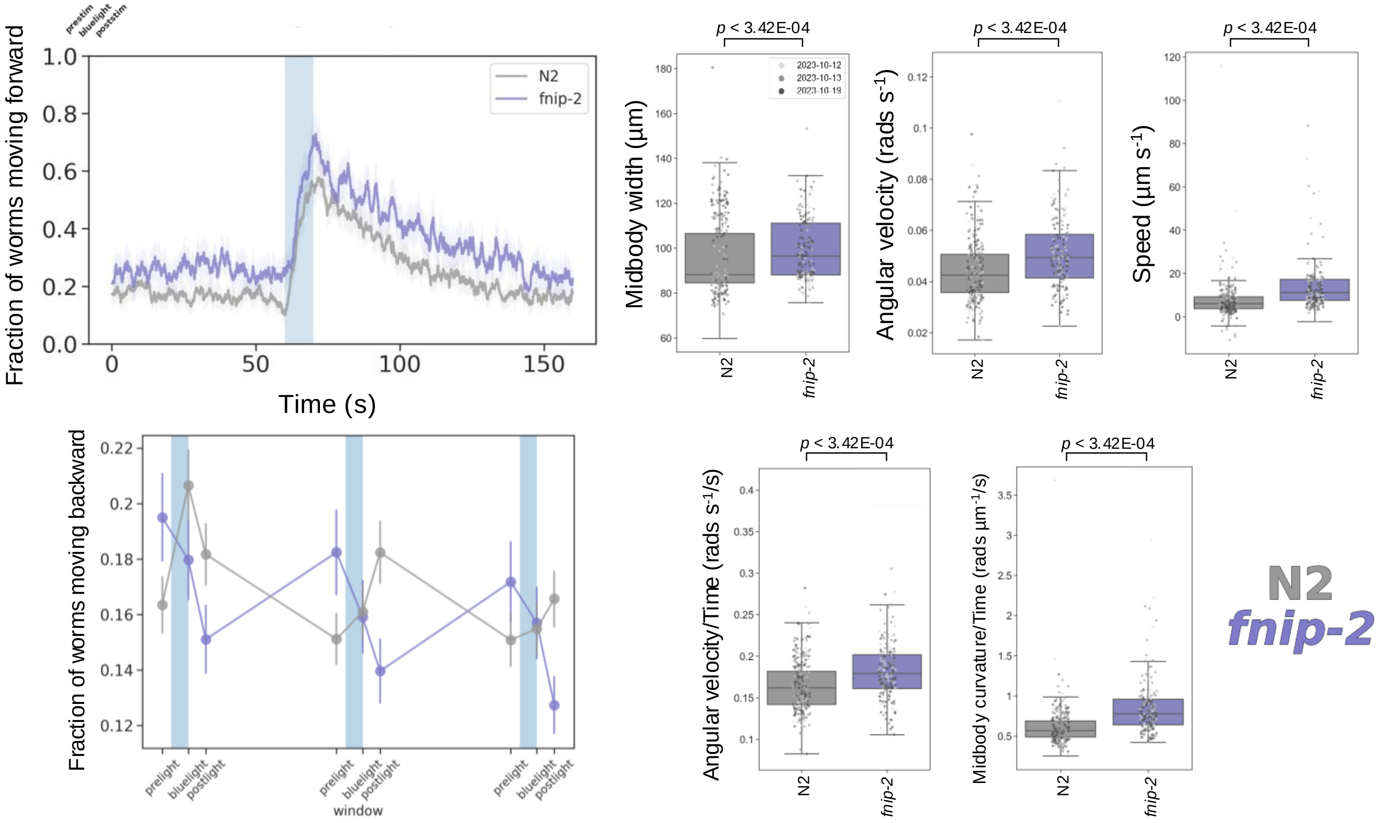

imb-2(syb6372)

Strain name: PHX6372 *imb-2*(*syb6372*)[D157N]
Wild-type gene length: 931bp
Mutation: D to N point mutation at amino acid position 157. Synonymous to D156N in TNPO2

C. elegans Description

Function: Nuclear import signal receptor activity; nuclear localisation sequence binding activity; protein import into nucleus

Expression: AWC^{ON}, AECL and AWCR sensory neurons

Previously reported phenotypes: Reduction of odour chemotaxis[24]

Human Orthologs:

TNPO2

Associated disease(s):
Intellectual developmental disorder with hypotonia; Impaired speech; Dysmorphic facies

Results

770/8289 significant features vs N2 ($p < 0.05$, block permutation t-test, 10000 permutations)

Key phenotype(s): No difference in morphology or locomotion; decreased angular velocity of head; increased curvature of midbody and tail; decreased head foraging (time derivative of angular velocity); decreased frequency of pausing (during stimulation with blue light; increased tail speed

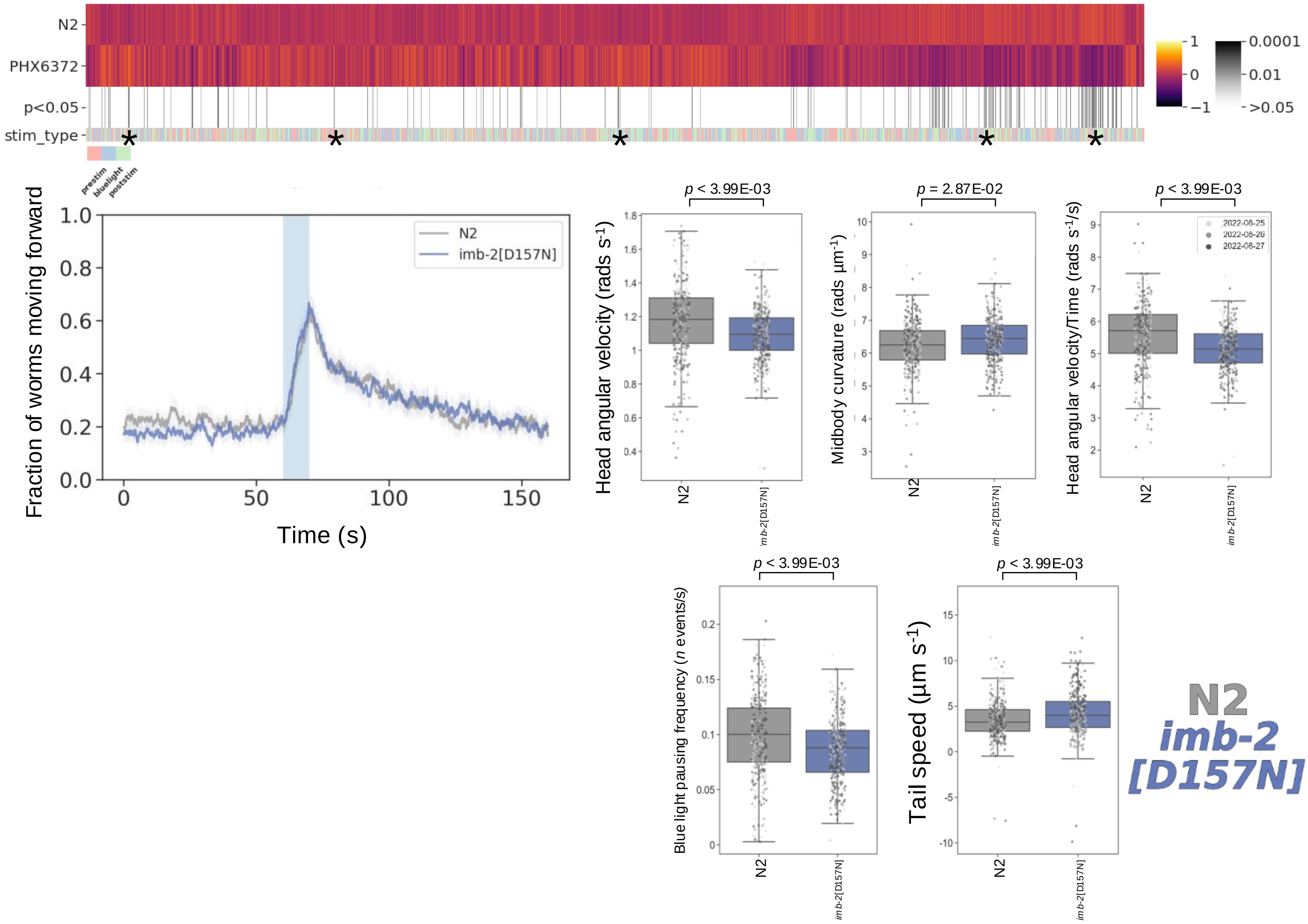

irk-1(syb5903)

Strain name: PHX5903
Wild-type gene length: 11931bp
Mutation: 11338bp deletion starting at nt position 168 (starting at beginning of exon 1)

C. elegans Description

Function: Enables inward rectifier potassium channel activity[25]; regulation of G protein-coupled receptor signalling[25]; regulation of egg-laying behaviour[25]; inhibition of HSNs by EGL-6 signaling[25]

Expression: Egg-laying apparatus; gonad; intestine; and neurons

Previously reported phenotypes: Mild defect in egg laying[26]

Human Orthologs:

KCJN2; KCNJ3 and KCNJ4

Associated disease(s):
Glucose metabolism disease; Heart conduction disease; Long QT syndrome

Results

3612/8289 significant features vs N2 ($p < 0.05$, block permutation t-test, 10000 permutations)

Key phenotype(s): Fatter; less active (high fraction of paused worms); decreased angular velocity of head while moving; lower frequency of head bends (time derivative of curvature); decreased; decreased head speed and overall speed of mutant

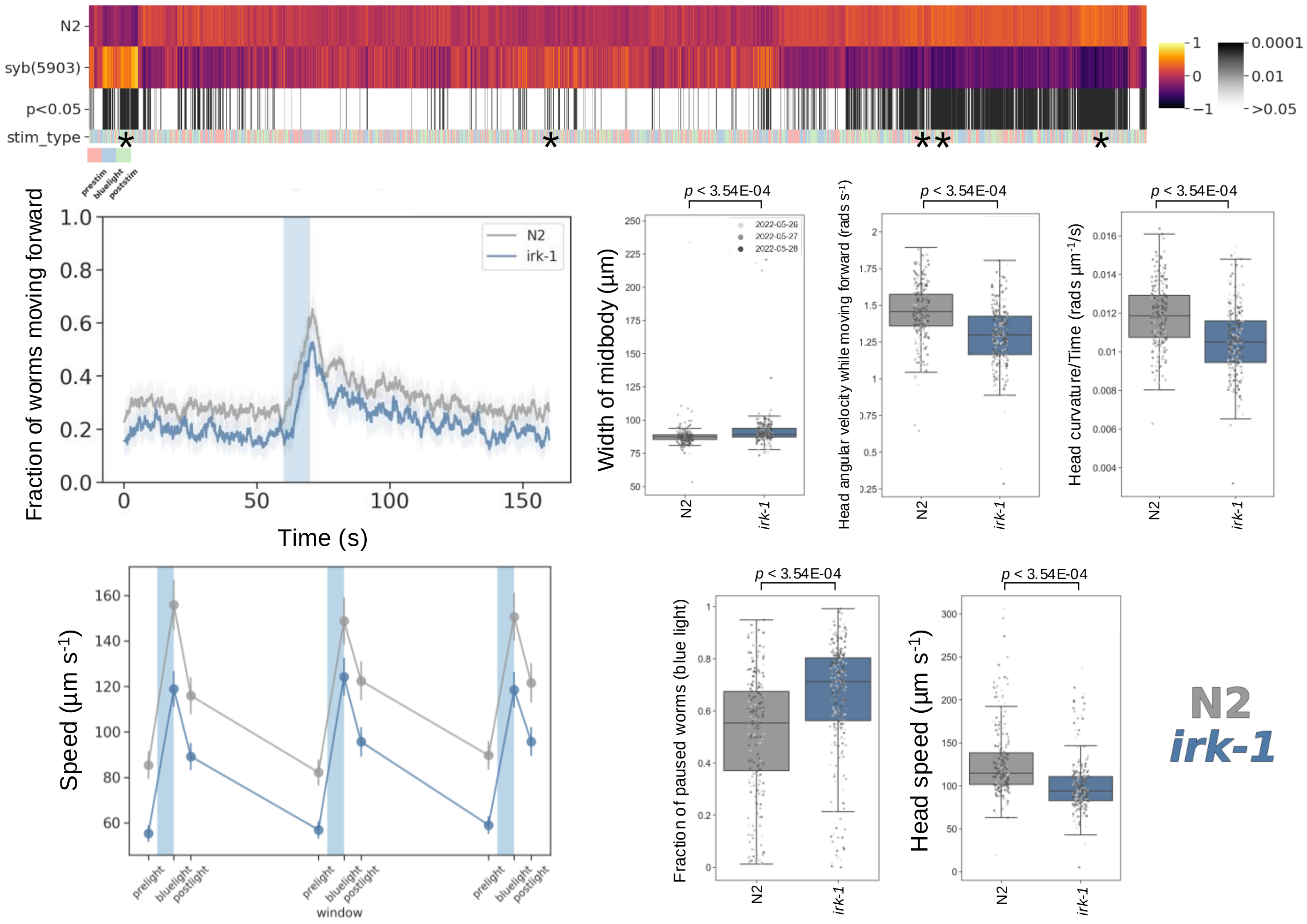

let-526(syb8759)

Strain name: PHX8759 *let-526*(*syb8759*)I/hT2[*bli-4*(*e937*)*let-?*(*q782*)*qIs48*] (I;III)
Wild-type gene length: 1003bp
Mutation: Balanced heterozygote containing a 9024bp deletion starting at nt position 91 (deletion of entire gene coding region)

C. elegans Description

<u>Function</u>: Enables DNA binding activity; contributes to nucleosome binding activity; involved in gonad and larval development; positive regulation of transcription by RNA polymerase II

<u>Expression</u>: Excretory cell; gonad; pharynx; tail precursor cell; and vulva

<u>Previously reported phenotypes</u>: Sterility (homozygotes)[27] ;defective migration of excretory cell precursor[27]; PVD structural abnormalities[28]; perturbations in dendritic patterning and cell fate specification[28]

Human Orthologs:

ARID1A and *ARID1B*

<u>Associated disease(s):</u>
Coffin-Siris syndrome; Carcinoma (multiple); Pre-eclampsia

Results

5008/8289 significant features vs N2 ($p < 0.05$, block permutation t-test, 10000 permutations)

Key phenotype(s): 6 hour developmental delay; fatter; no difference in baseline locomotion, but attenuated photophobic escape response; decreased duration of movement in response to blue light; decreased angular velocity (all body segments); decreased curvature of head and neck; slower; lower frequency of head bends/foraging (time derivative of head curvature)

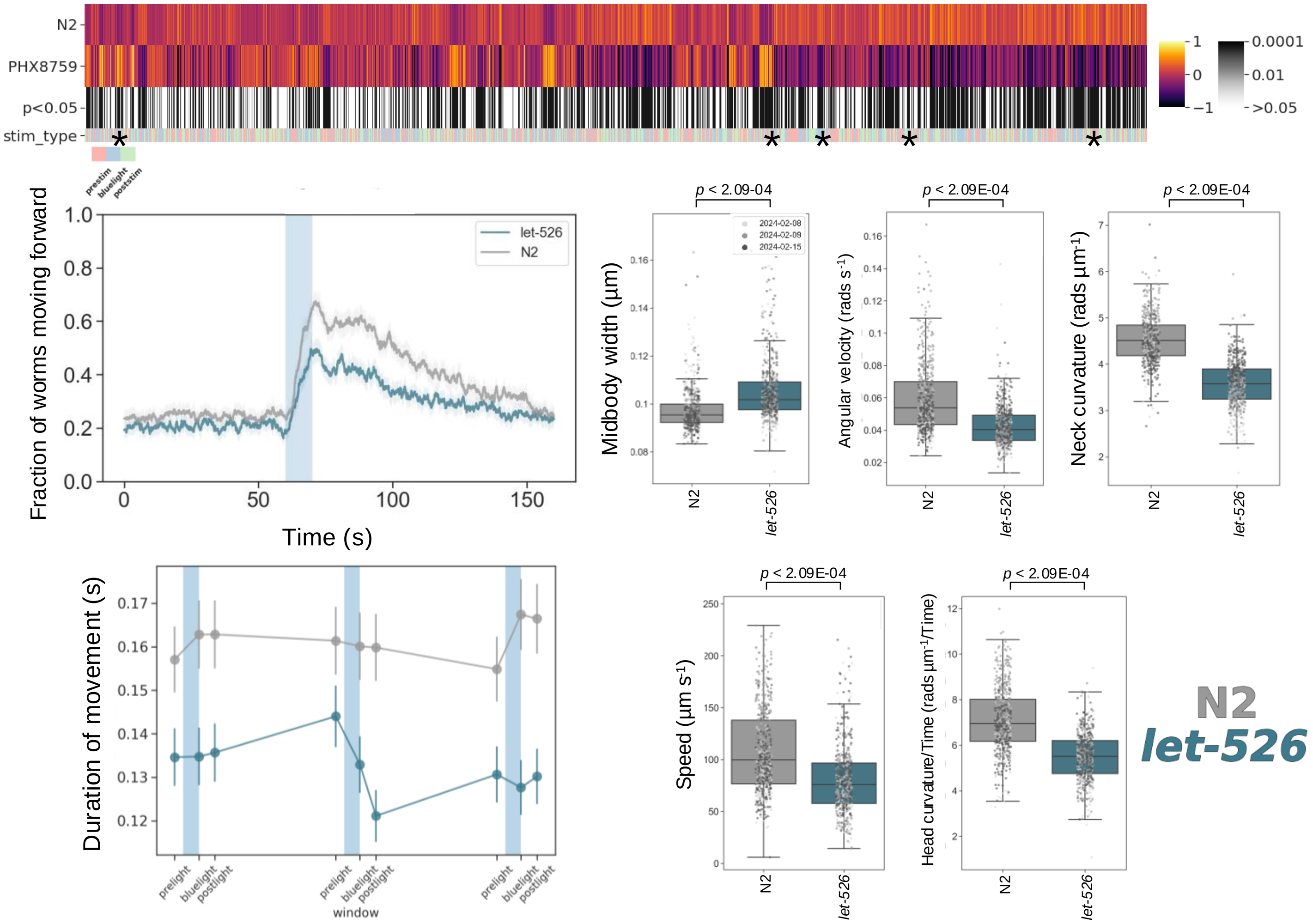

ncap-1(syb4935)

Strain name: PHX4935
Wild-type gene length: 4624bp
Mutation: 4252bp deletion starting at nt position 166 (starting at first half of exon 1)

C. elegans Description

Function: Predicted to be involved in vesicle-mediated transport negative regulators of the AP2 clathrin adaptor complex[29]

Expression: Nerve ring

Previously reported phenotypes: None

Human Orthologs:

NECAP1 and NECAP2

Associated disease(s):
Developmental and epileptic encephalopathy (21)

Results

1826/8289 significant features vs N2 ($p < 0.05$, block permutation t-test, 10000 permutations)

Key phenotype(s): Shorter; decreased angular velocity, but increased curvature, of all body segments; less active; attenuated photophobic escape response; slower

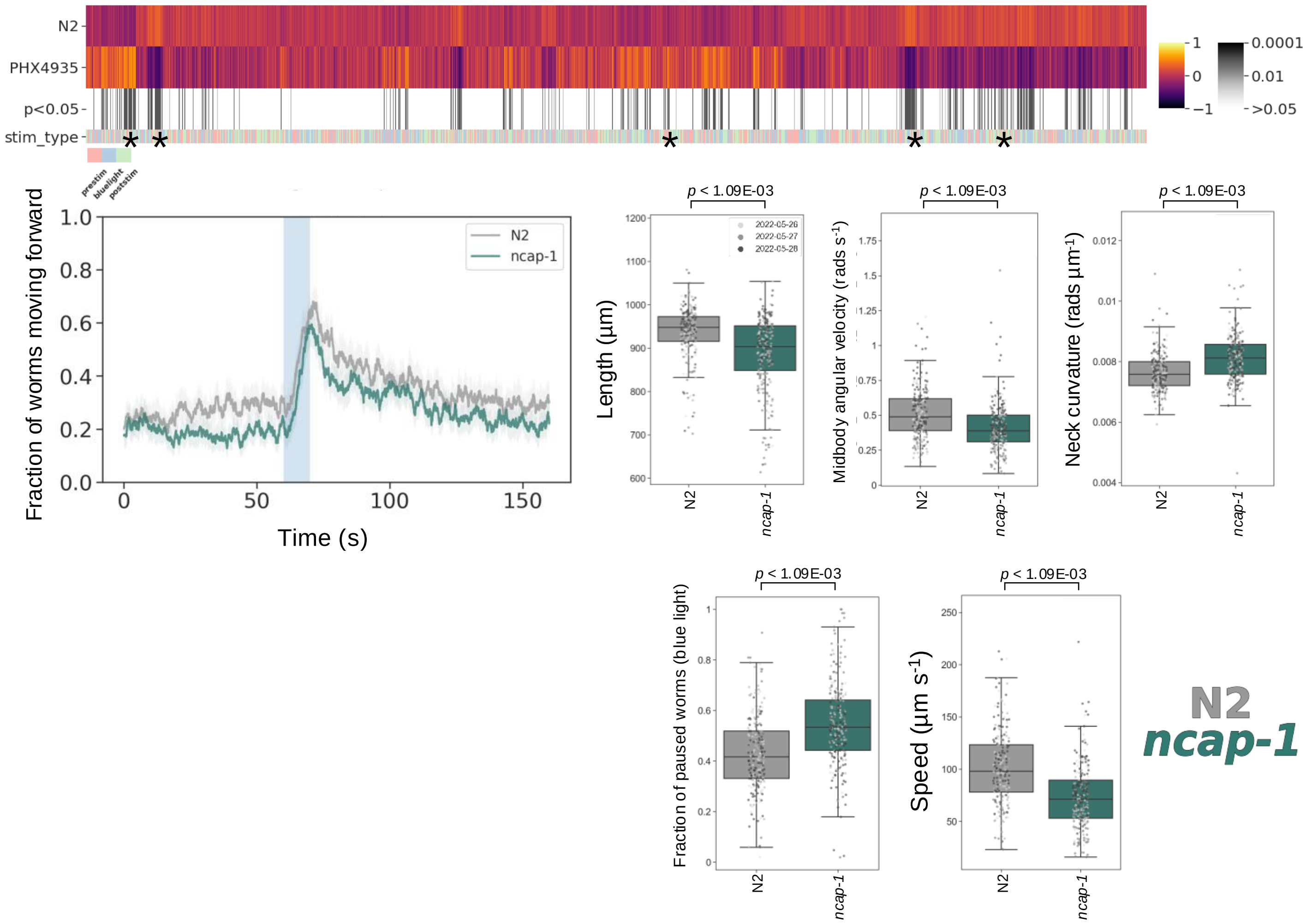

odr-8(syb4940)

Strain name: PHX4940
Wild-type gene length: 3171bp
Mutation: 2585bp deletion starting at nt position 39 (starting from first half of exon 1)

C. elegans Description

Function: Enables deUFMylase activity; positive regulation of chemotaxis; protein localisation to non-motile cilium

Expression: Amphid and phasmid neurons

Previously reported phenotypes: AWA odorant chemotaxis defective[30]; diacetyl chemotaxis defective[31]; aldicarb hypersensitivity[31]

Human Orthologs:

UFSP2

Associated disease(s):
Early infantile epileptic encephalopathy; Developmental epileptic encephalopathy; Beukes hip dysplasia

Results

4557/8289 significant features vs N2 ($p < 0.05$, block permutation t-test, 10000 permutations)

Key phenotype(s): Developmental delay (7h); less active; shorter; decreased curvature (for all body segments); slower; decreased duration of forward movement, but a greater and more sustained backwards escape response to photophobic stimulation

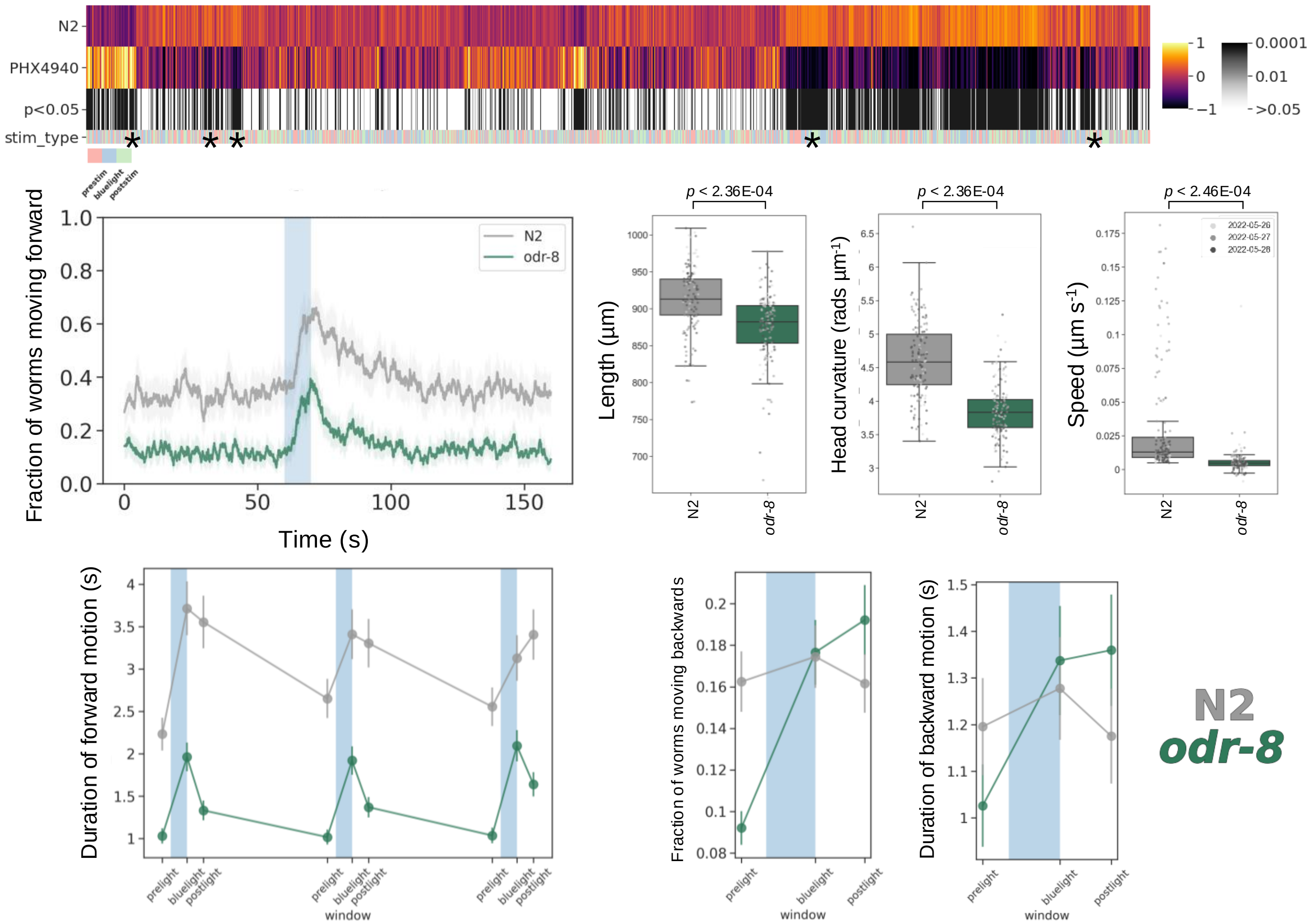

pacs-1(syb7634)

Strain name: PHX7634 *pacs-1*(*syb7634*)[E205K]
Wild-type gene length: 1166bp
Mutation: E to K amino acid substitution at position 205- Synonymous to E209K mutation in human PACS2

C. elegans Description

Function: Involved in protein localisation to plasma membrane

Expression: Excretory cell; intestine; neurons; pharynx; vulva

Previously reported phenotypes: Aldicarb resistance[32]

Human Orthologs:

PACS1 and PACS2

Associated disease(s):
Schuurs-Hoeijmakers Syndrome; Developmental and epileptic encephalopathy (66); PACS1 syndrome; Seizures; Developmental delay; Intellectual disability; Autism

Results

3667/8289 significant features vs N2 ($p < 0.05$, block permutation t-test, 10000 permutations)

Key phenotype(s): Less active; decreased angular velocity; decreased curvature of head, neck, hips and midbody; lower frequency of body bends (time derivative of midbody curvature); decreased speed; attenuated photophobic escape response

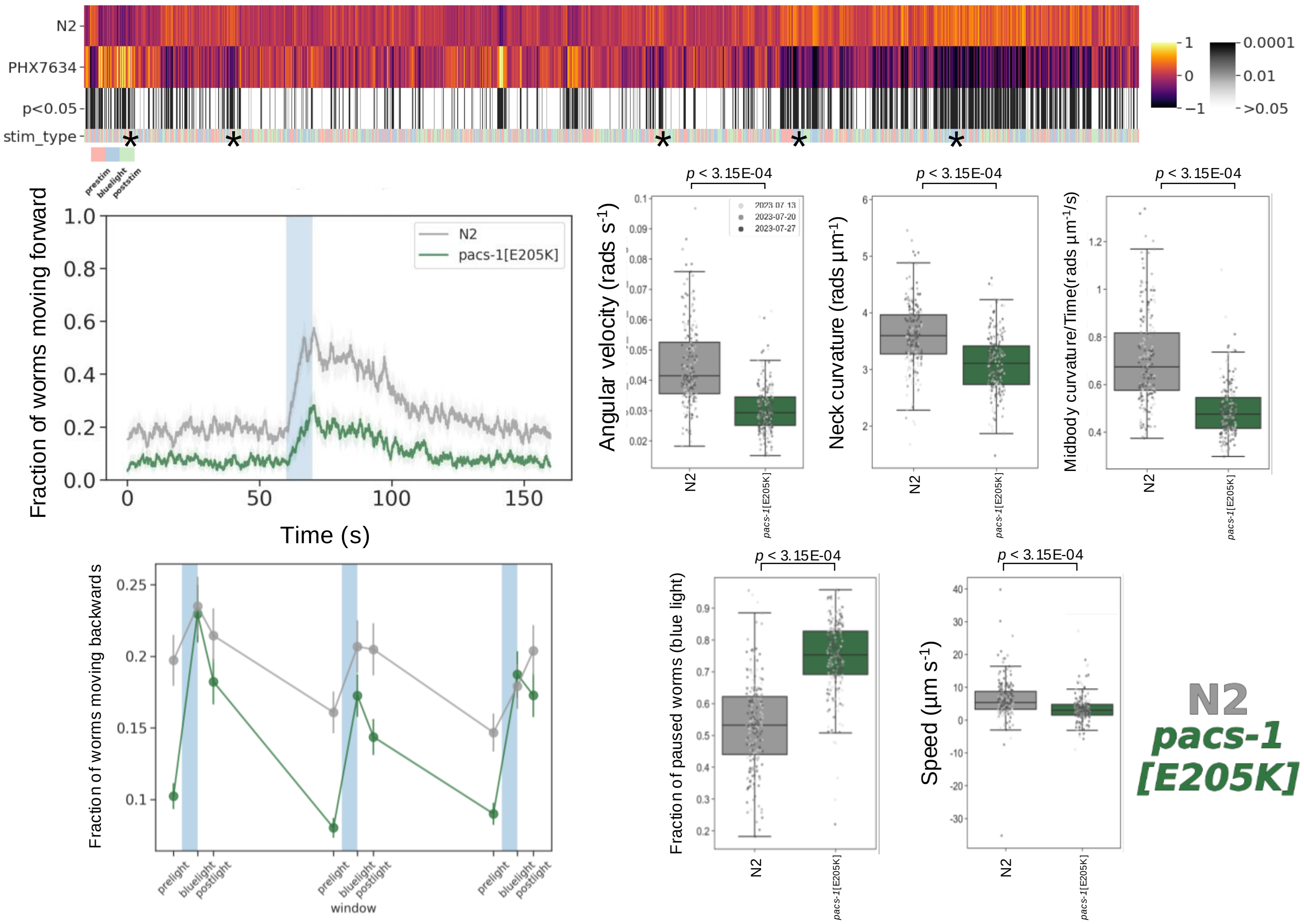

pde-1(syb7700)

Strain name: PHX7700
Wild-type gene length: 7830bp
Mutation: 7095bp deletion starting at nt position 40 (deletion of entire gene coding region)

C. elegans Description

Function: Enables 3',5'-cyclic-AMP phosphodiesterase, 3',5'-cyclic-GMP phosphodiesterase and calmodulin binding activity; negative regulation of intracellular signal transduction; determination of adult lifespan; response to alkaline pH

Expression: Head; AFDL and AFDR neurons

Previously reported phenotypes: Isothermal tracking behaviour variant[33]; odorant adaptation variant[34]

Human Orthologs:

PDE1A and *PDE1B*

Associated disease(s):
Autosomal dominant nonsyndromic deafness (74); Cardiac disorders (multiple); Impotence; Schizophrenia

Results

3502/8289 significant features vs N2 (p < 0.05, block permutation t-test, 10000 permutations)

Key phenotype(s): Shorter; fatter; decreased curvature (all body segments); lower frequency of body bends (time derivative of midbody curvature); less active during baseline recordings; shorter duration of movement in response to blue light; no difference in baseline speed, but decreased speed during photophobic escape response

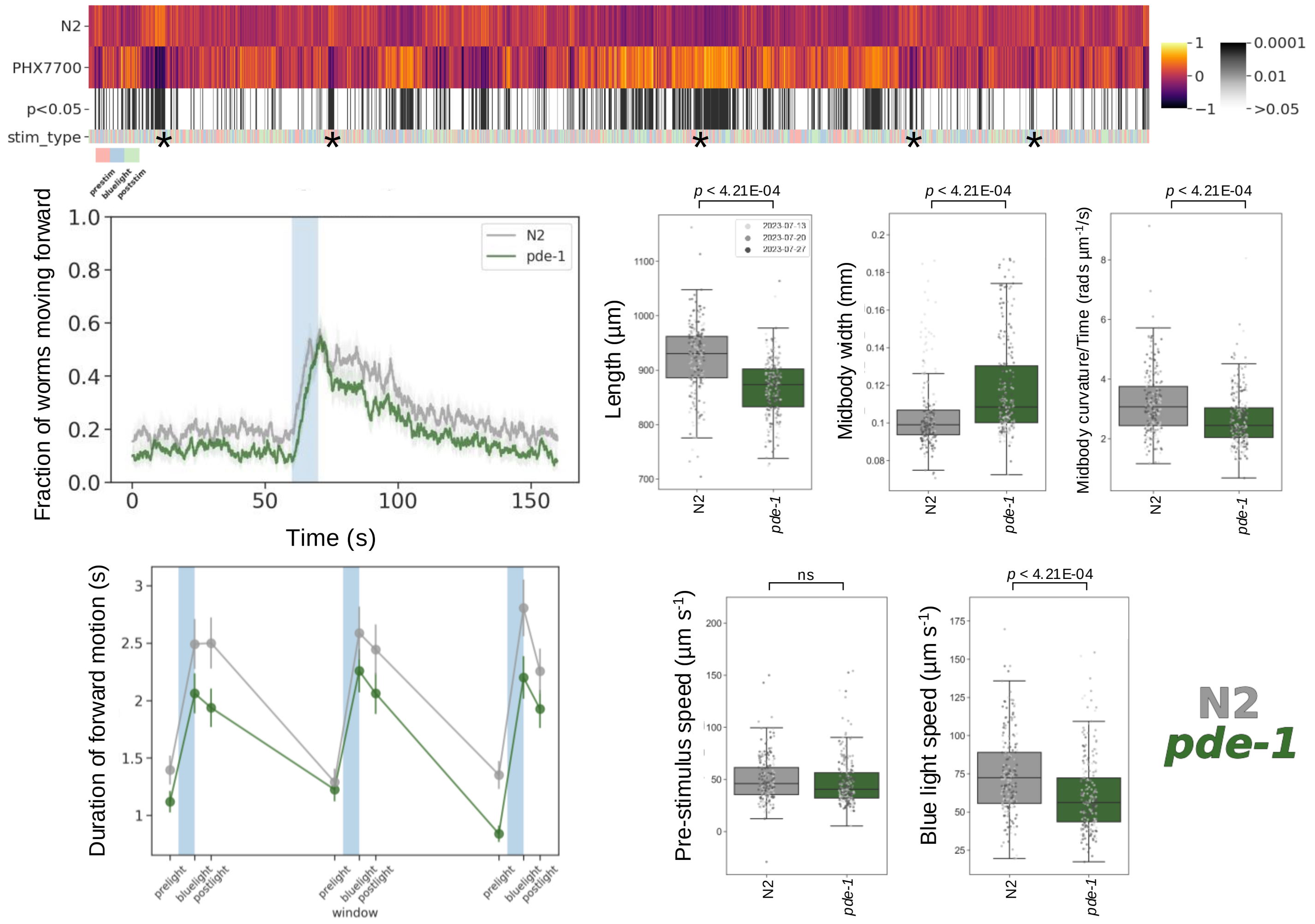

pde-5(syb7506)

Strain name: PHX7506
Wild-type gene length: 4025bp
Mutation: 4025bp deletion of the entire gene

C. elegans Description

Function: Predicted to enable 3',5'-cyclic-GMP phosphodiesterase activity and cGMP-stimulated cyclic-nucleotide phosphodiesterase activity; negative regulation of cGMP-mediated signalling ;determination of adult lifespan; response to alkaline pH

Expression: AFDL and AFDR neurons

Previously reported phenotypes: Isothermal tracking behaviour variant[33]; odorant adaptation variant[34]

Human Orthologs:

PDE10A

Associated disease(s):
Schizophrenia; Huntington's disease; Dyskinesia; Striatal degeneration; Addiction

Results

3492/8289 significant features vs N2 (p < 0.05, block permutation t-test, 10000 permutations)

Key phenotype(s): Longer and fatter; most behavioural phenotypes affect the head only (location of gene expression), including: decreased angular velocity, curvature and speed, with lower frequency of head bends (time derivative of curvature); general locomotion is unaffected

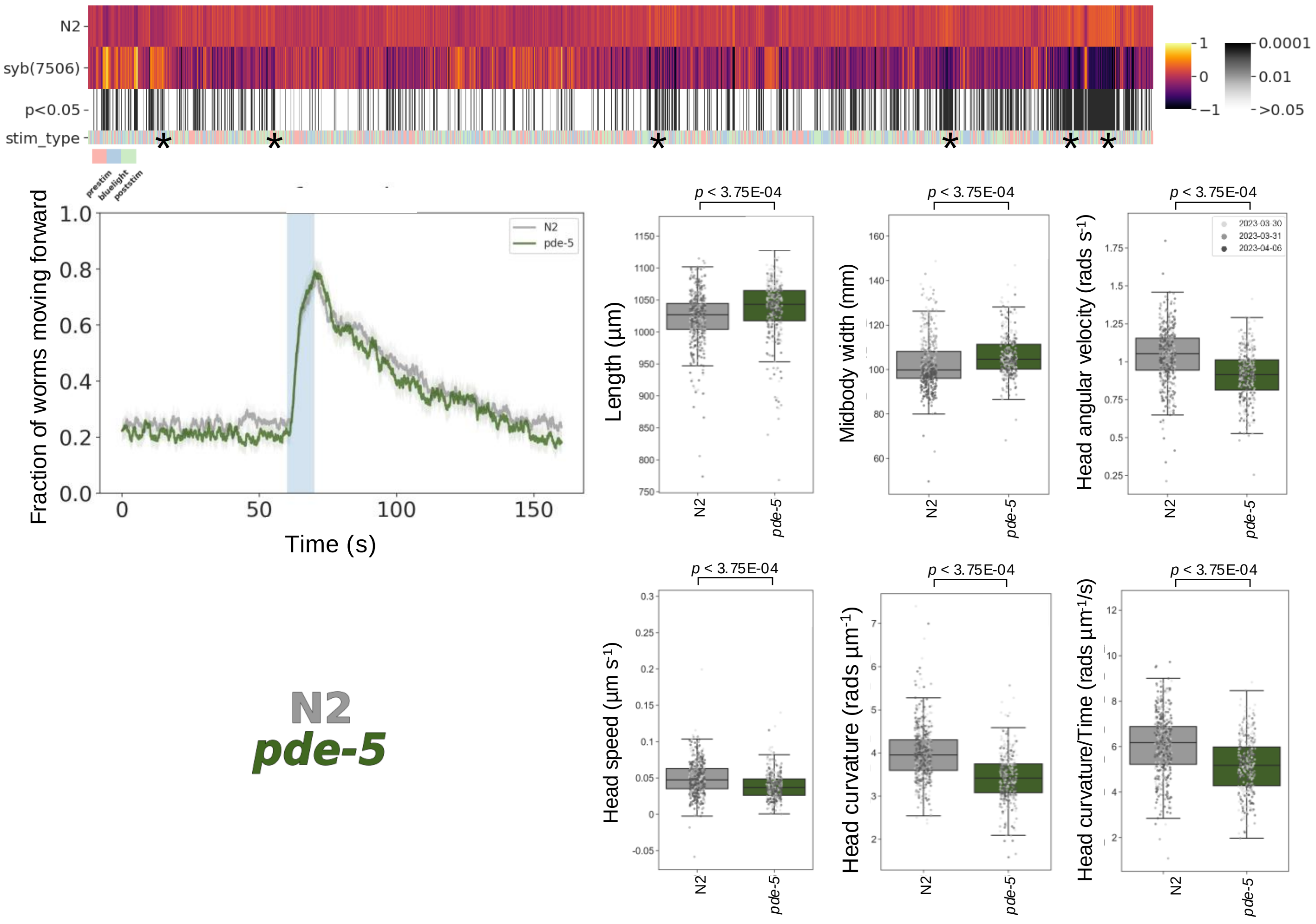

pmp-4(syb7777)

Strain name: PHX
Wild-type gene length: 3047bp
Mutation: 3047bp deletion of the entire gene

C. elegans Description

<u>Function</u>: Enables ATP binding activity; ATPase-coupled transmembrane and long-chain fatty acid transporter activity; involved in the fatty acid catabolic process, long-chain fatty acid import into peroxisomes and peroxisome organisation

<u>Expression</u>: Peroxisomal membrane (predicted)

<u>Previously reported phenotypes</u>: Decreased cilia length[35]; dye filling defect[35]; defective nonanone chemotaxis[35]

Human Orthologs:

ABCD1 and *ABCD2*

<u>Associated disease(s):</u>
Adrenoleukodystrophy

Results

3329/8289 significant features vs N2 ($p < 0.05$, block permutation t-test, 10000 permutations)

Key phenotype(s): Shorter; increased angular velocity of tail and neck; lower frequency of tail bends (time derivative of tail angular velocity); decreased curvature of neck and midbody; no statistically significant changes in locomotion

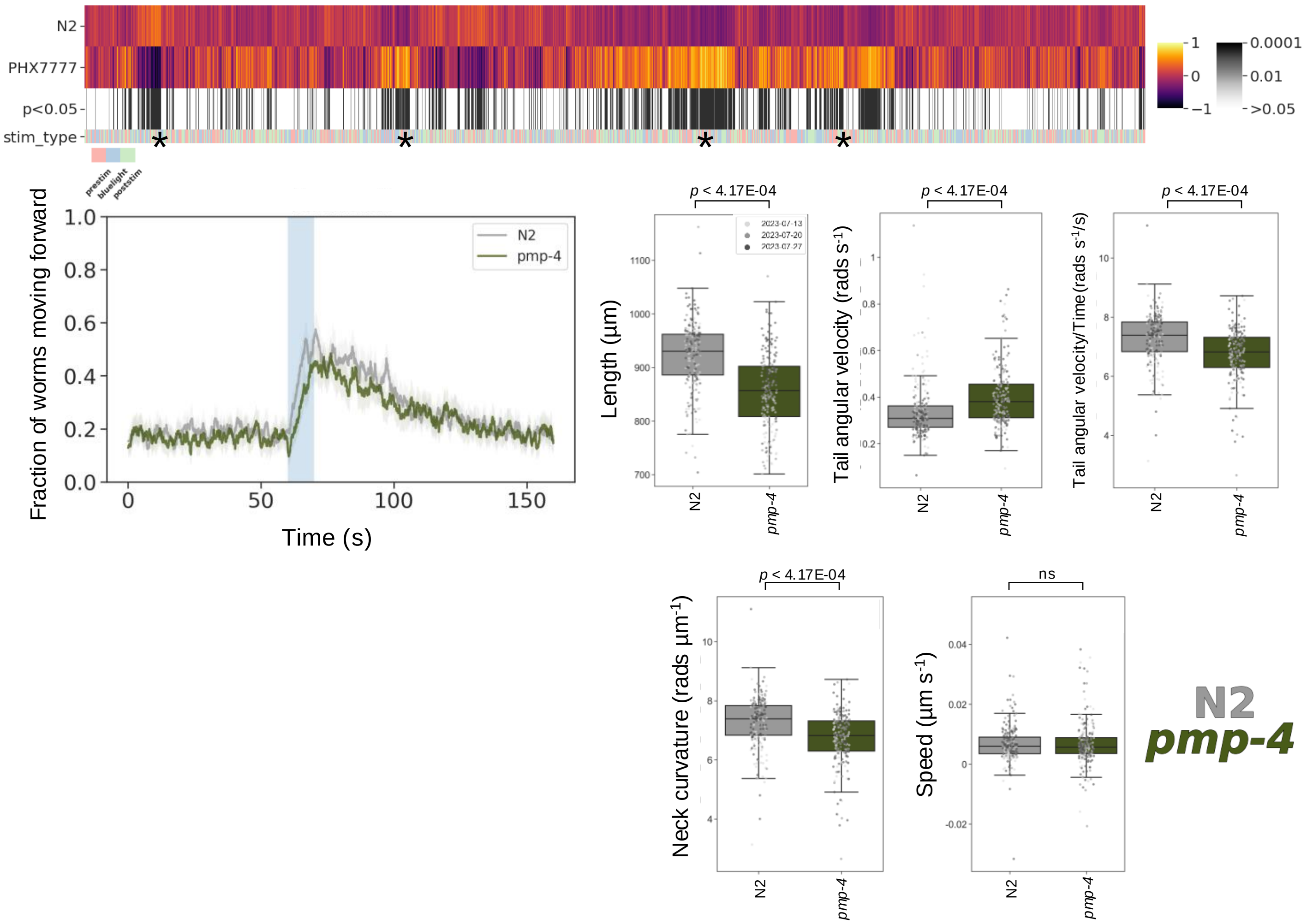

R10E11.6(syb7507)

Strain name: PHX7507
Wild-type gene length: 2858bp
Mutation: 2488bp deletion starting at nt position 26 (deletion of entire gene coding region)

C. elegans Description

<u>Function</u>: Interacts with several genes, including: *daf-12*, *daf-16* and *gld-1* (function unknown)

<u>Expression</u>: AVK and NSM neurons; anterior hypodermis and germline

<u>Previously reported phenotypes</u>: Neuron degeneration[36]; necrotic cell death variant[37]

Human Orthologs:

SYNRG

<u>Associated disease(s):</u>
Chromosome 17Q12 Deletion Syndrome; Mixed Phenotype Acute Leukemia

Results

5405/8289 significant features vs N2 (*p* < 0.05, block permutation t-test, 10000 permutations)

Key phenotype(s): Shorter; less active, even during stimulation with blue light; decreased angular velocity and curvature (of all body segments); lower frequency of head bends (time derivative of head curvature); and decreased overall speed

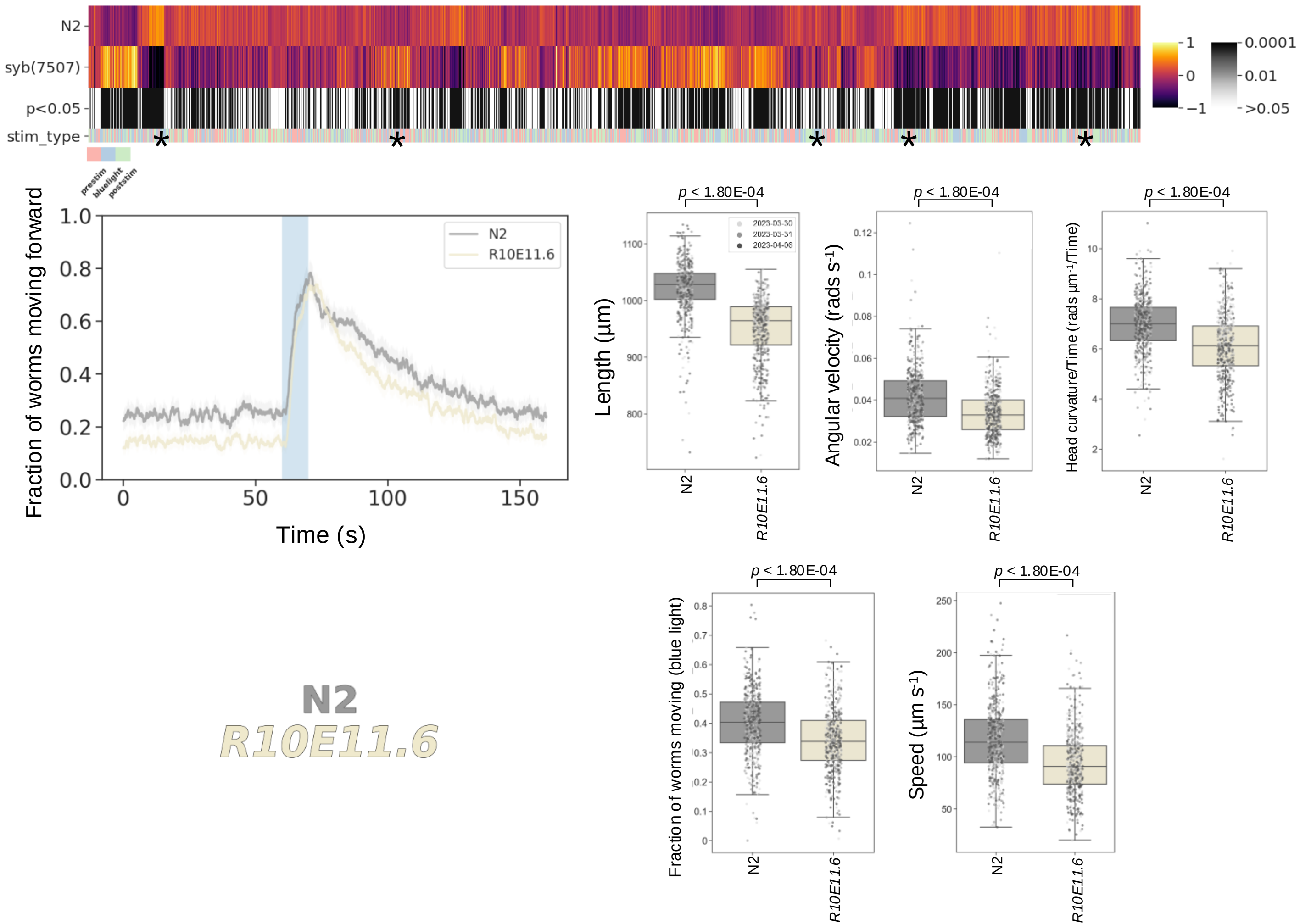

rpy-1(syb5027)

Strain name: PHX5027
Wild-type gene length: 3603bp
Mutation: 3054bp deletion starting at nt position 26 (deletion of entire gene coding region)

C. elegans Description

Function: Enables acetylcholine receptor binding activity; involved in cholinergic synaptic transmission and positive regulation of neuromuscular synaptic transmission

Expression: Neurons

Previously reported phenotypes: Levamisole resistance[38]; increased fat content[39]

Human Orthologs:

RAPSN

Associated disease(s):
congenital myasthenic syndrome (11); Fetal akinesia deformation sequence syndrome (2)

Results

3661/8289 significant features vs N2 ($p < 0.05$, block permutation t-test, 10000 permutations)

Key phenotype(s): Shorter; decreased angular velocity; increased curvature of midbody and tail; decreased head speed and lower frequency of head bends (time derivative of head curvature); no difference in overall locomotion of the mutant

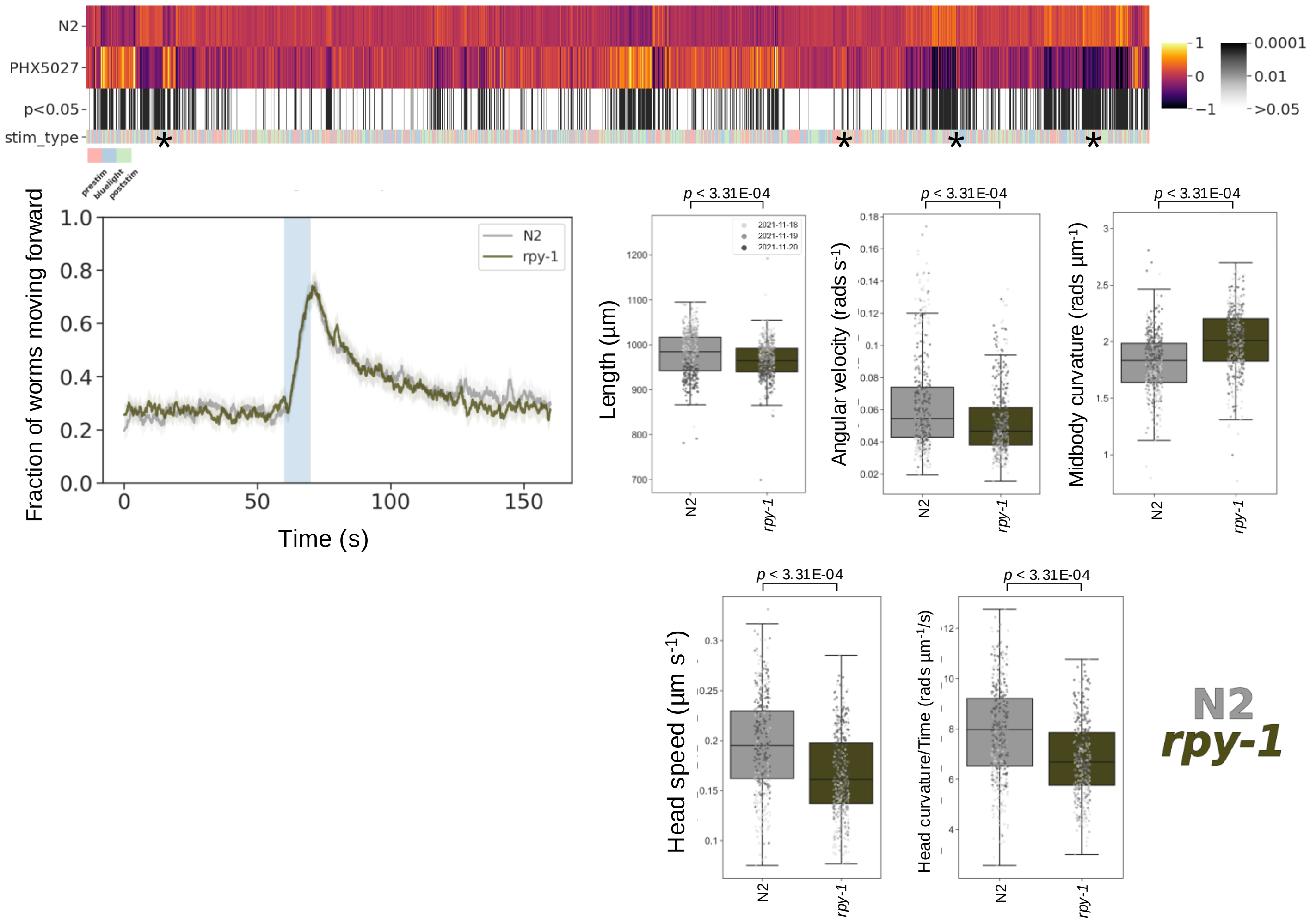

sam-4(syb6765)

Strain name: PHX6765
Wild-type gene length: 1305bp
Mutation: 757bp deletion starting at nt position 234 (half of exon 1 and remaining coding regions)

C. elegans Description

<u>Function</u>: Enables guanyl-nucleotide exchange factor activity; positive regulator of anterograde synaptic vesical transport (*via* enhancement of UNC-104 recruitment/activation on synaptic vesicles)[42] and pharyngeal pumping[43]

<u>Expression</u>: Nerve ring

<u>Previously reported phenotypes</u>: Dysregulated synaptic vesicle transport[40]; uncoordinated locomotion[41]

Human Orthologs:

BORCS5

<u>Associated disease(s):</u>
Hermansky-Pudlak syndrome; Colorectal adenocarcinoma; Polymicrogyria; Isolated Corpus Callosum Agenesis

Results

3201/8289 significant features vs N2 ($p < 0.05$, block permutation t-test, 10000 permutations)

Key phenotype(s): Shorter; decreased angular velocity, curvature, speed and acceleration of head; no statistically significant difference in other locomotion related features

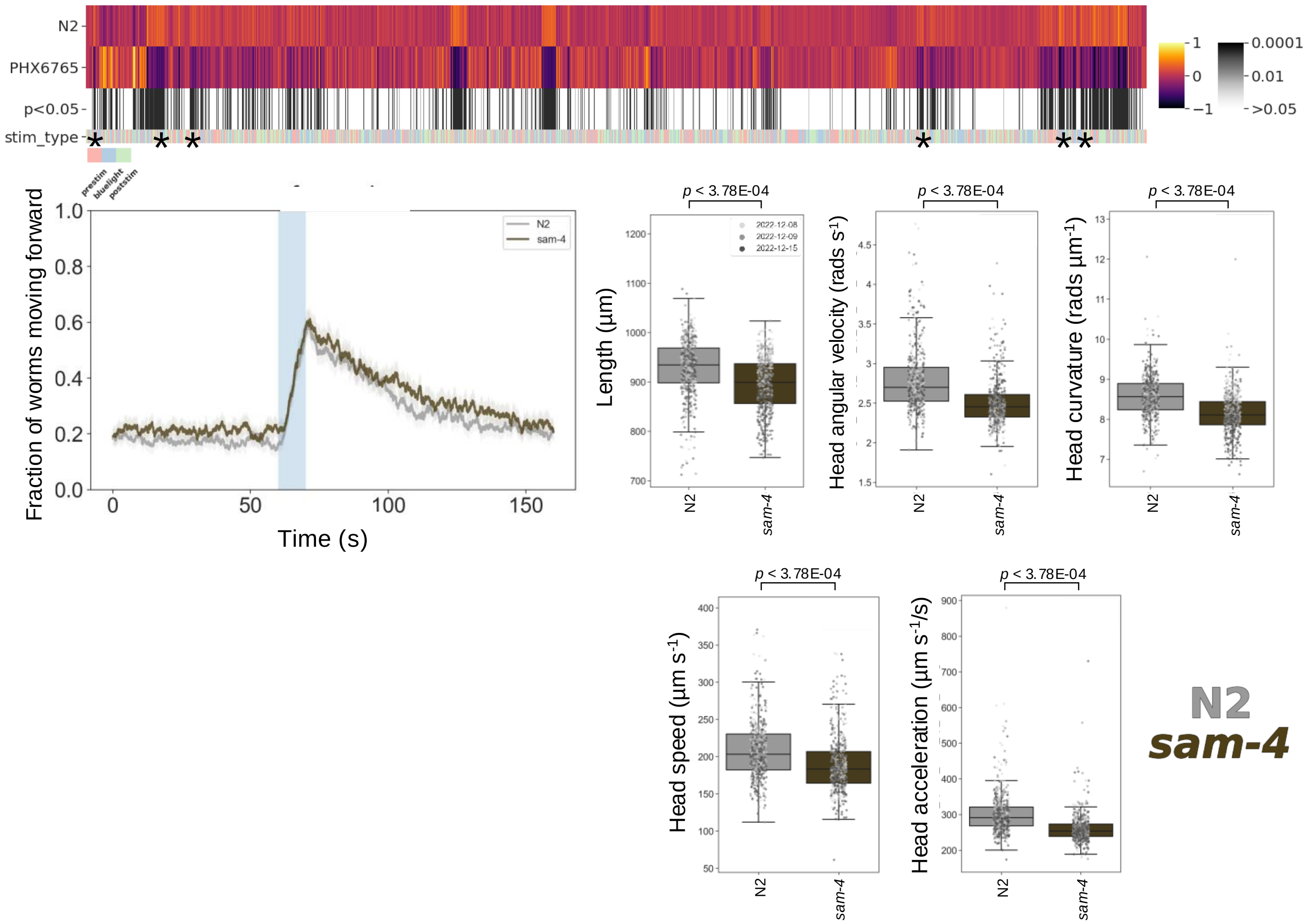

sec-31(syb6825)

Strain name: PHX6825
Wild-type gene length: 5815bp
Mutation: 5815bp deletion starting at nt position 40 (deletion of entire gene coding region)

C. elegans Description

Function: Predicted to enable structural molecule activity and be involved in COPII-coated vesicle cargo loading, endoplasmic reticulum organisation and intracellular protein transport

Expression: Endoplasmic reticulum exit site

Previously reported phenotypes: Reduced brood size[42]

Human Orthologs:

SEC31A

Associated disease(s):
Halperin-Birk syndrome; Soft tissue sarcoma

Results

4236/8289 significant features vs N2 ($p < 0.05$, block permutation t-test, 10000 permutations)

Key phenotype(s): Longer; decreased angular velocity of head and tail; decreased curvature of head and neck; lower frequency of midbody bends (time derivative of midbody curvature); no difference in locomotion features

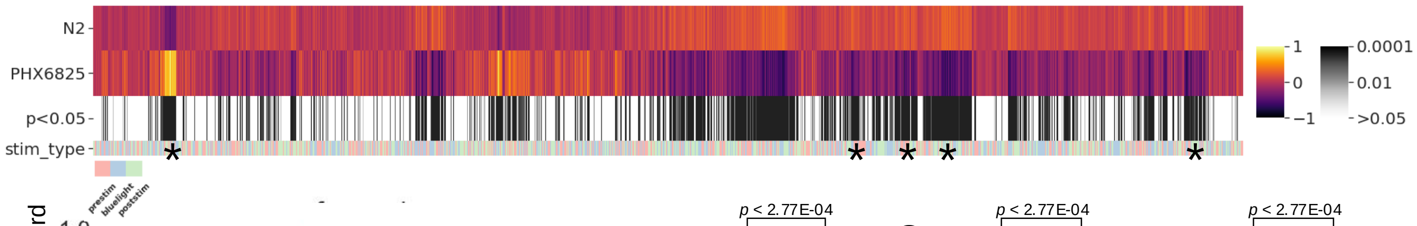

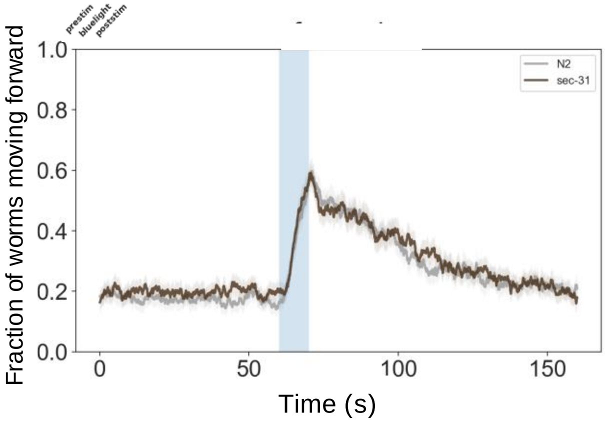

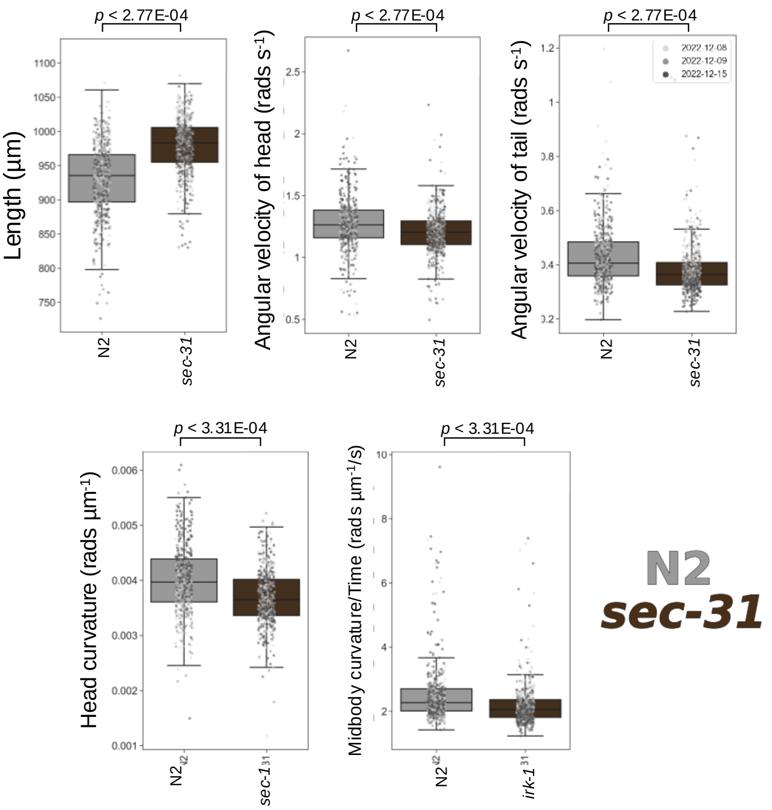

shl-1(syb5907)

Strain name: PHX5907
Wild-type gene length: 5589bp
Mutation: 5166bp deletion starting at nt position 91 (start of exon 1 to end of exon 5)

C. elegans Description

Function: Predicted to enable transient outward potassium channel activity; involved in potassium ion transmembrane transport

Expression: Body wall musculature; muscle cell neurons; pharynx; tail

Previously reported phenotypes: Reduced male mating efficiency[43]; reduction of peak transient (electrical) current of myocytes[44]

Human Orthologs:

KCND2 and *KCND3*

Associated disease(s):
Brugada syndrome (9); Spinocerebellar ataxia (types 19 and 22)

Results

289/8289 significant features vs N2 ($p < 0.05$, block permutation t-test, 10000 permutations)

Key phenotype(s): Fatter; decreased angular velocity of head while moving; decreased head acceleration (time derivative of head speed); attenuated backing, but sustained forward response, upon stimulation with blue light

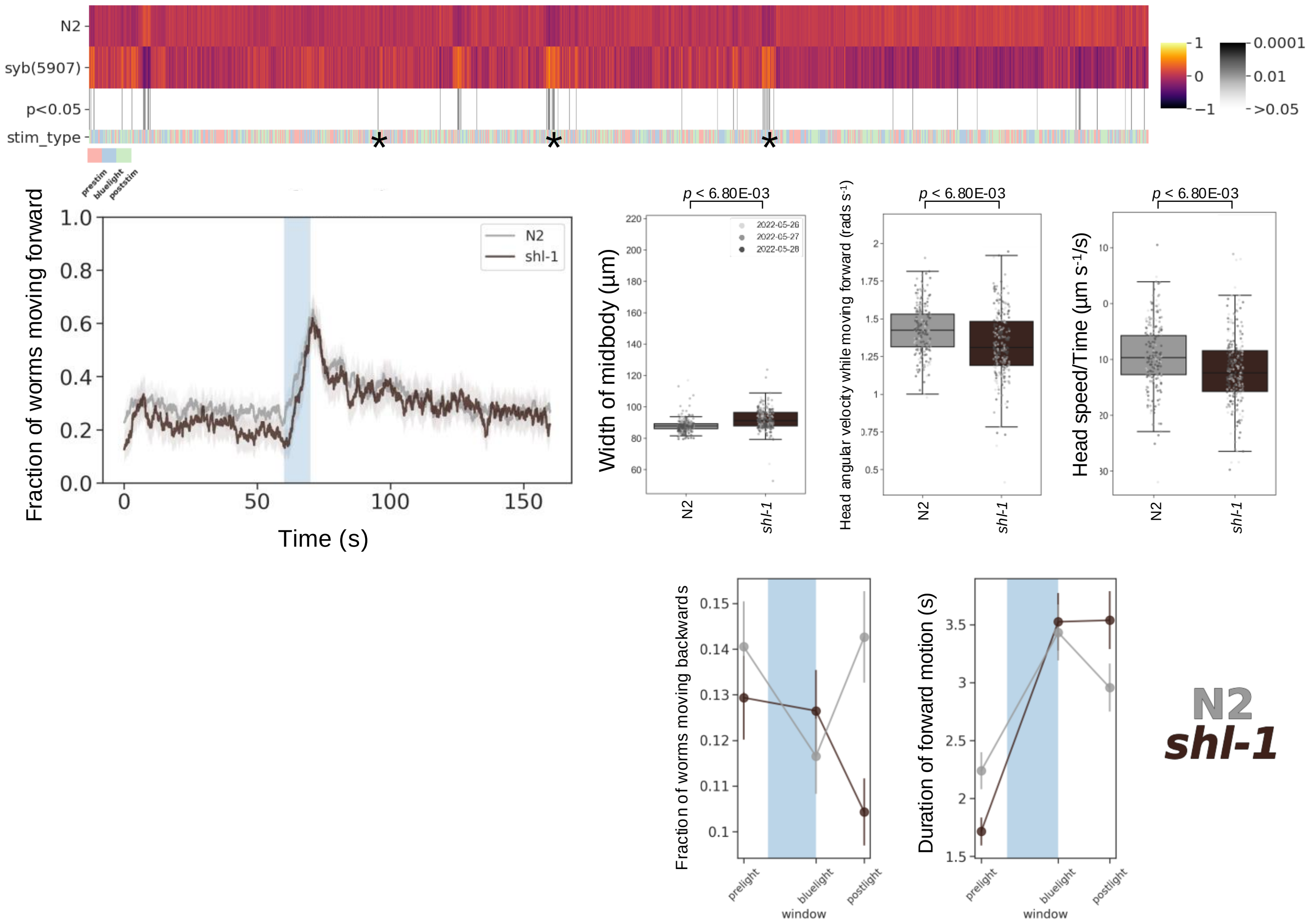

smc-3(fq180)

Strain name: *smc-3*((*fq180*)[K115E] III)
Wild-type gene length: 4003bp
Mutation: K to E amino acid substitution position 115. Synonymous to K114E mutation in human *SMC3*

C. elegans Description

Function: Enables cohesion loader activity and double-stranded DNA binding activity; involved in mitotic sister chromatid cohesion

Expression: Chromatin and cohesion complex

Previously reported phenotypes: Embryonic lethality (for genetic knockdown)[45]; reduced brood size[46]; meiosis variant[46]

Human Orthologs:

SMC3

Associated disease(s):
Cornelia de Lange syndrome; Hepatocellular carcinoma; Intellectual disability

Results

5685/8289 significant features vs N2 ($p < 0.05$, block permutation t-test, 10000 permutations)

Key phenotype(s): Longer; increased midbody curvature; decreased curvature and angular velocity of the head; less active during baseline tracking, with no difference in photophobic escape response; increased duration of pausing following stimulation with blue light

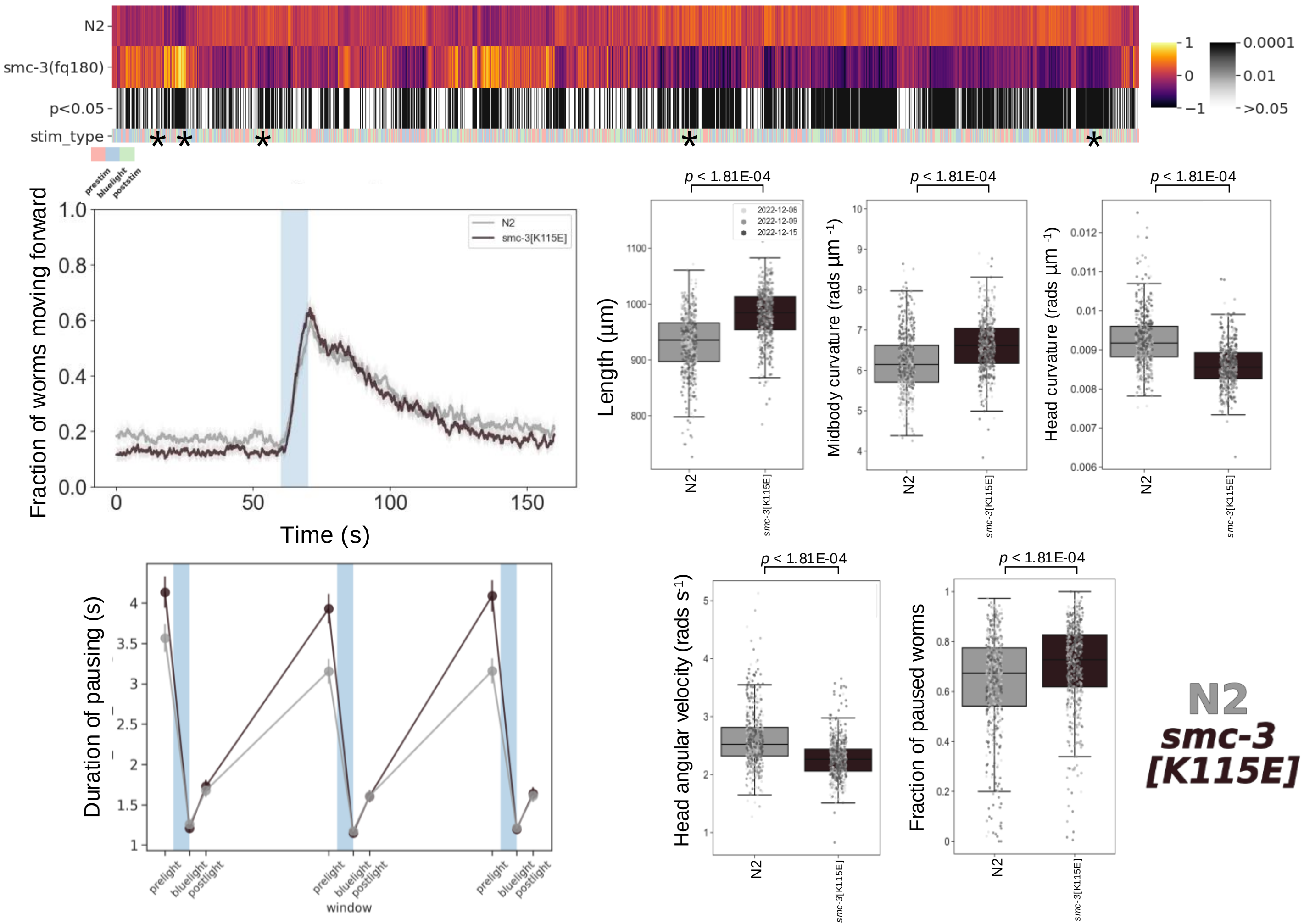

tmem-222(syb4882)

Strain name: PHX4882
Wild-type gene length: 657bp
Mutation: 544bp deletion starting at nt position 16 (deletion of entire gene coding region)

C. elegans Description

<u>Function</u>: Predicted to be an integral membrane component

<u>Expression</u>: Uncharacterised

<u>Previously reported phenotypes</u>: None

Human Orthologs:

TMEM222

<u>Associated disease(s):</u>
Neurodevelopmental disorder with motor and speech delay; Non-specific syndromic intellectual disability

Results

68/8289 significant features vs N2 (*p* < 0.05, block permutation t-test, 10000 permutations)

Key phenotype(s): Increased midbody curvature

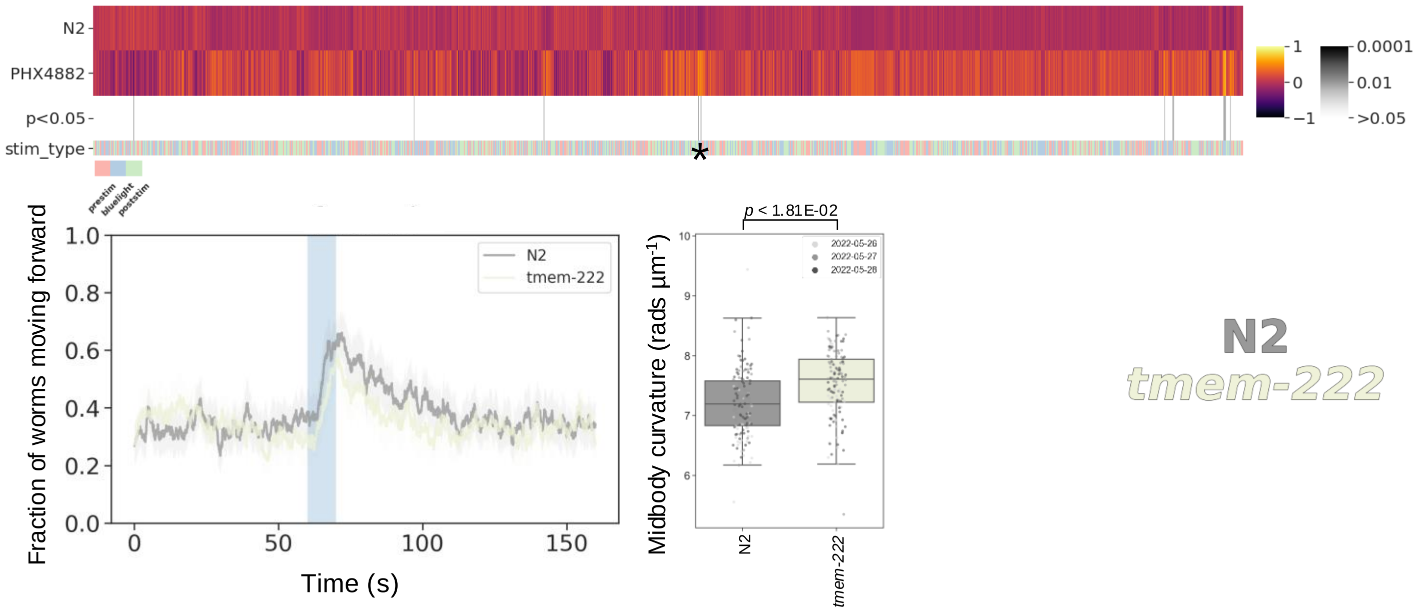

vps-50(syb6653)

Strain name: PHX6653
Wild-type gene length: 5137bp
Mutation: 5111bp deletion starting at nt position 201 (deletion of entire gene coding region)

C. elegans Description

<u>Function</u>: Predicted to enable SNARE binding activity; positive regulation of dense core granule transport; regulation of locomotory behaviour

<u>Expression</u>: Cytosol and EARP complex

<u>Previously reported phenotypes</u>: Increased duration of egg retention[47]; lack of slowing in response to food[47]

Human Orthologs:

VPS50

<u>Associated disease(s):</u>
Neurodevelopmental disorder with microcephaly; Seizures; Neonatal cholestasis; Pontocerebellar hypoplasia, Type 2E

Results

6952/8289 significant features vs N2 ($p < 0.05$, block permutation t-test, 10000 permutations)

Key phenotype(s): Less active during baseline recordings; strong, but short-lived (increased paused duration), forward photophobic escape response; sustained backward escape response; shorter; decreased angular velocity and curvature (for all body segments); slower

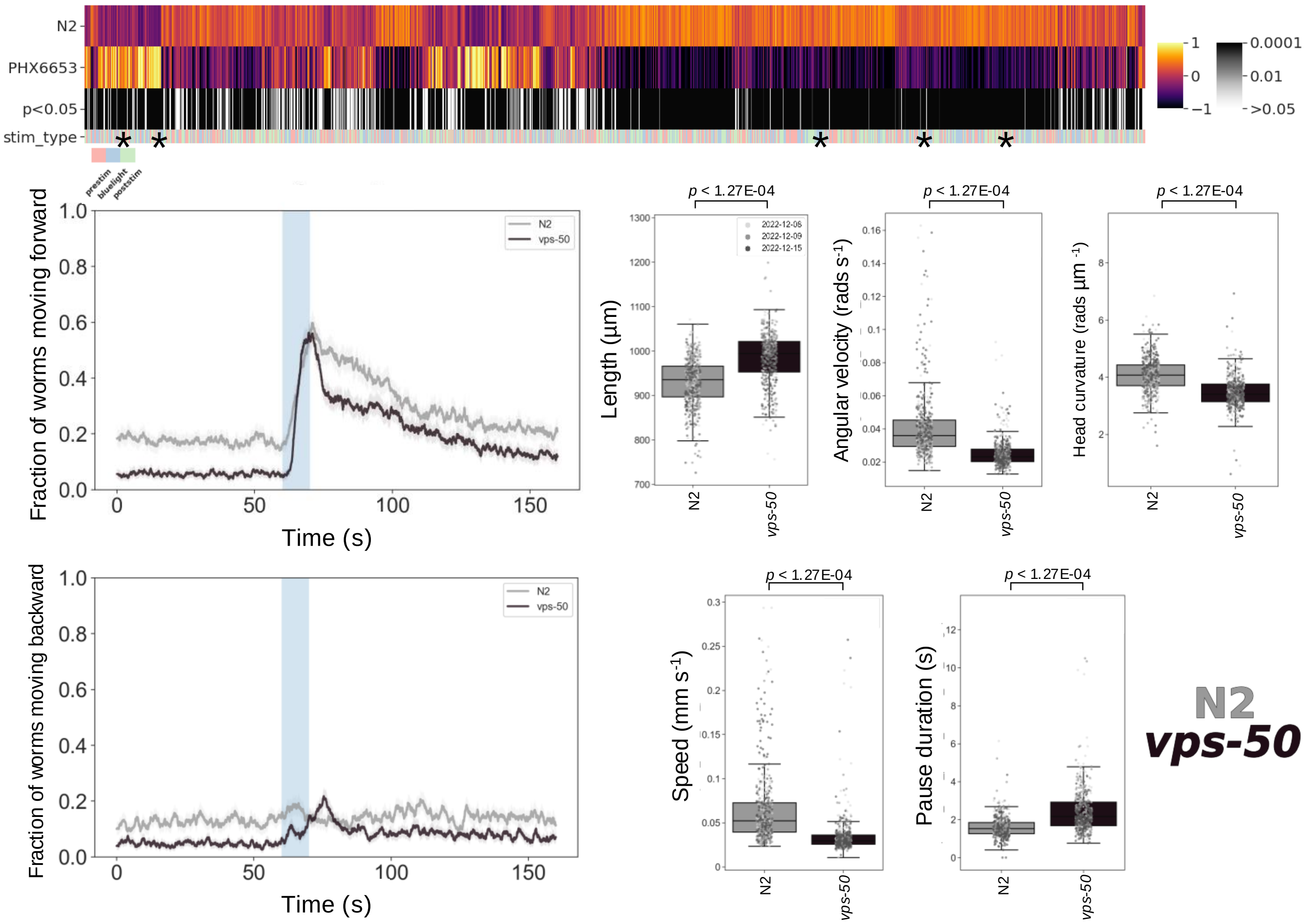

Y47D9A.1(syb7612)

Strain name: PHX7612 *Y47D9A.*1(*syb7612*)[R298W]
Wild-type gene length: 983bp
Mutation: R to W amino acid substitution position 298 - Synonymous to R318W mutation inhuman GMPPA

C. elegans Description

<u>Function</u>: Predicted to enable transferase activity

<u>Expression</u>: Cytoplasm (predicated)

<u>Previously reported phenotypes</u>: None

Human Orthologs:

GMPPA

<u>Associated disease(s):</u>
Alacrimia; Achalasia; Impaired intellectual development syndrome

Results

3704/8289 significant features vs N2 ($p < 0.05$, block permutation t-test, 10000 permutations)

Key phenotype(s): Attenuated photophobic escape response; slow moving; shorter; decreased angular velocity; increased frequency of body bends (time derivative of midbody curvature)

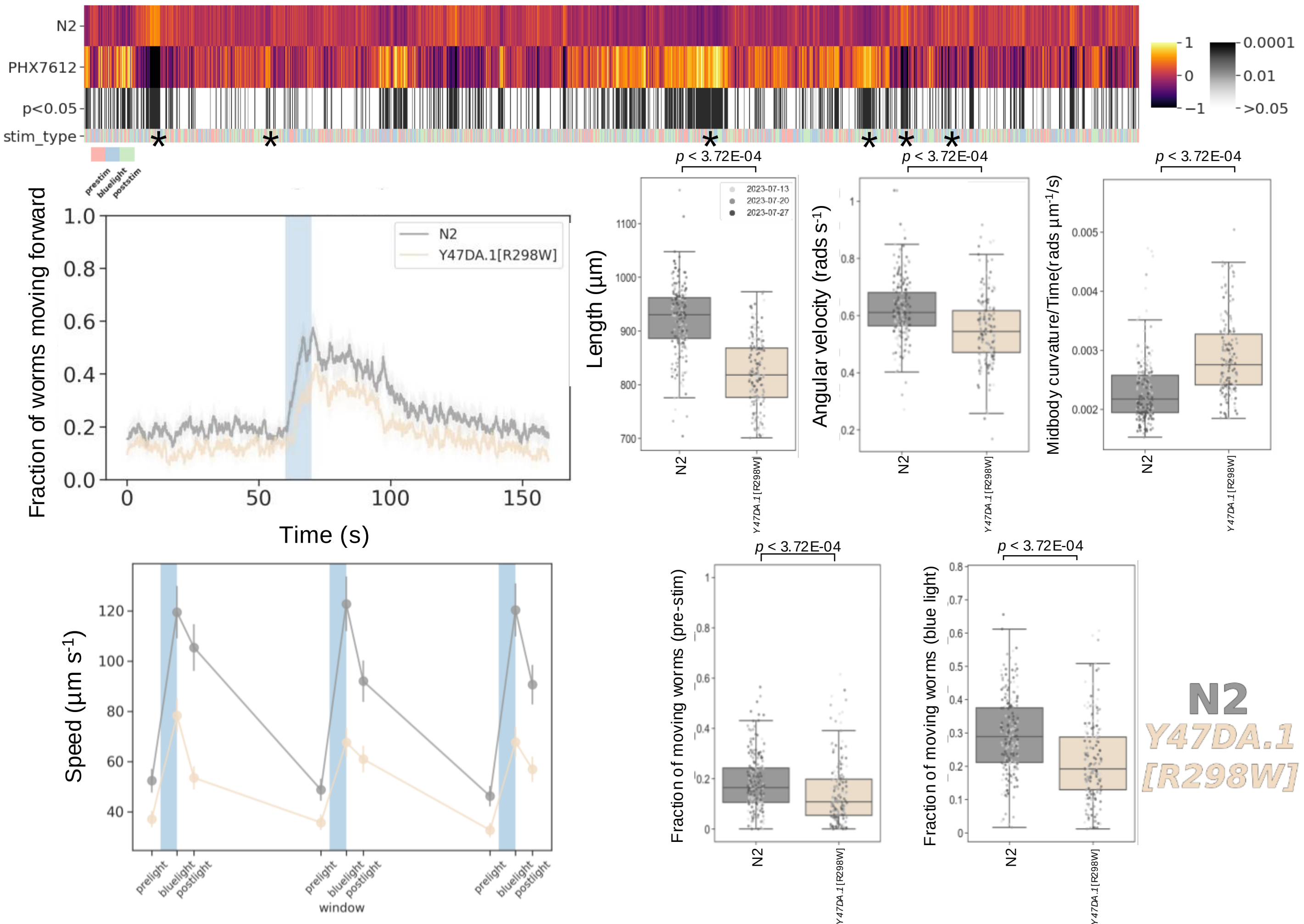

Supplement: Supplementary file 1 — Additional file 1. Strain-specific cards. Detailing: phenotype summaries; the molecular characteristics; previously reported phenotypes (if available) and human disease(s) associated with each strain studied. [file 12915_2025_2368_MOESM1_ESM.pdf]
