# Supplementary figures and images for: High-throughput behavioural phenotyping of 25 C. elegans disease models including patient-specific mutations
Source: BMC Biol. 2025 Sep 26;23:281. doi: 10.1186/s12915-025-02368-8 (PMC12465487; doi:10.1186/s12915-025-02368-8)

**A**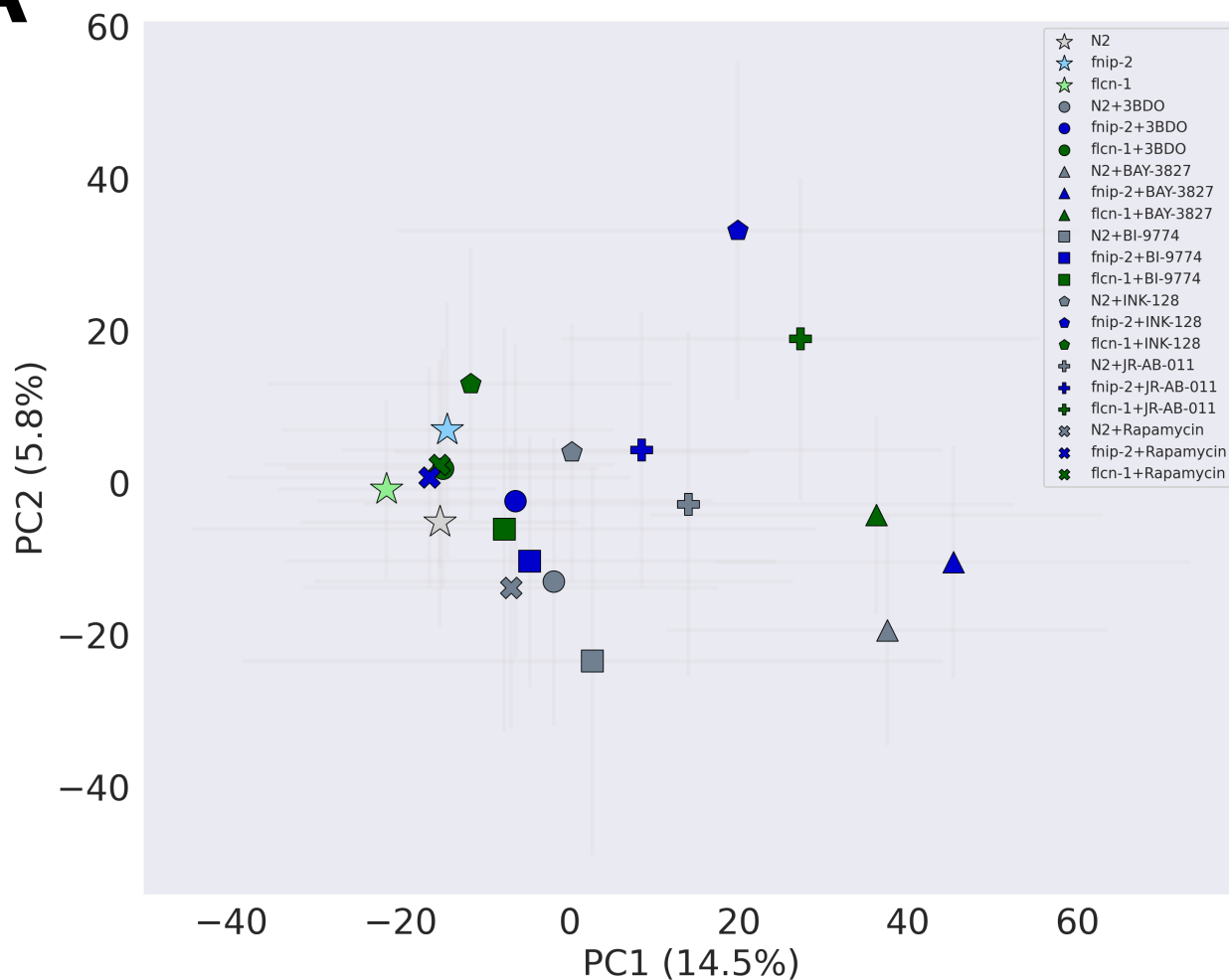**B**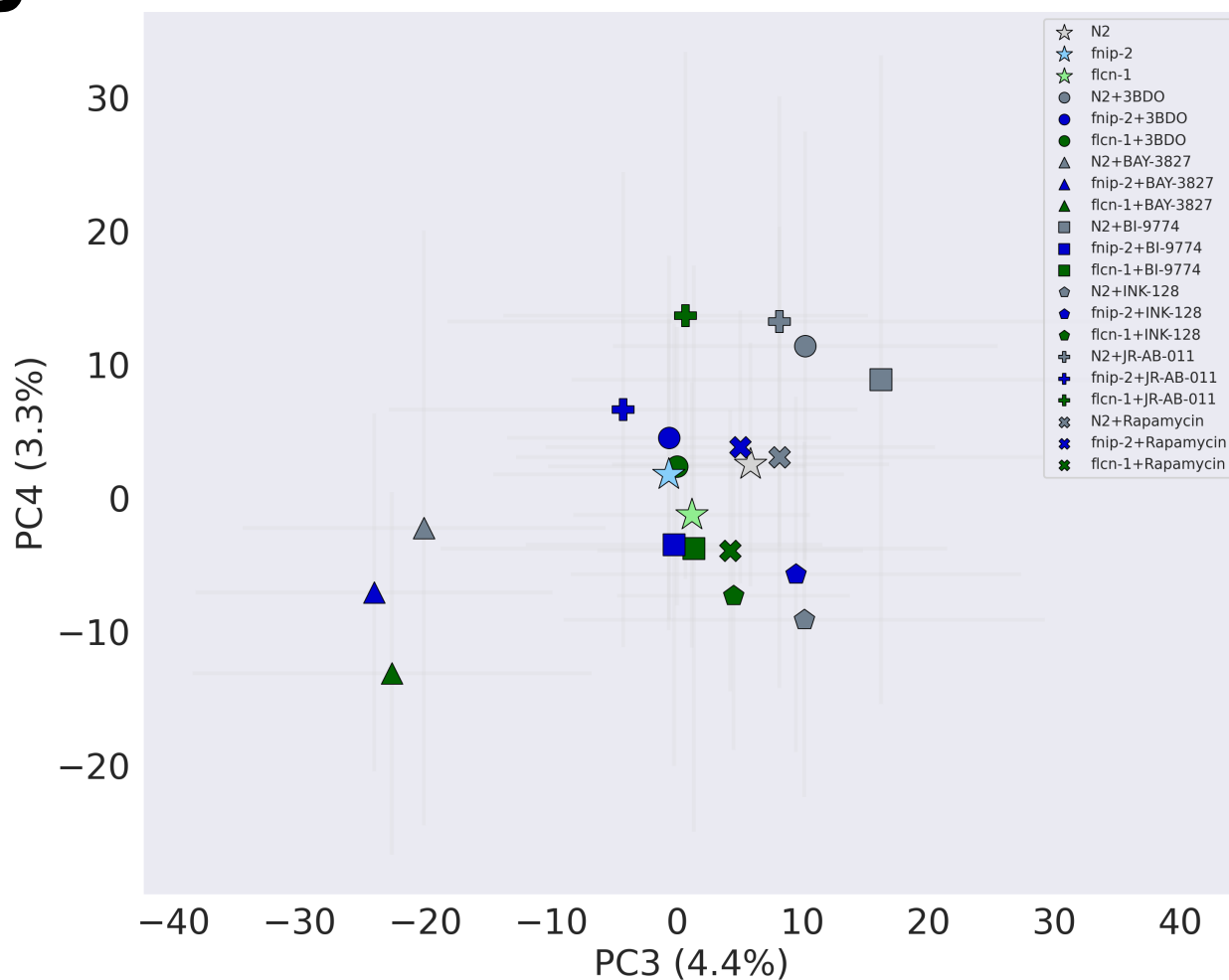

Supplement: Supplementary file 2 — Additional file 2. Principal component analysis of AMPK and mTOR inhibition or activation on folliculin mutant phenotypes. PCA of the folliculin disease model mutants and wild-type strains treated with DMSO only (stars) and the same strains treated with 100 µM of each compound (different shaped, darker makers) for 4 h prior to tracking (n > 17). Variance explained by the components is denoted in brackets and error bars represent standard deviation. (A) First 2 principal components of the entire behavioural feature set extracted by Tierpsy, (B) third and further principal components. PCA analysis reveals that treatment with the AMPK or mTOR inhibitor/activators tested did not restore the mutant phenotypes to wild-type. However, treatment with the different compounds moves the wild-type and mutant strains in the same direction within phenotypic space. [file 12915_2025_2368_MOESM2_ESM.pdf]

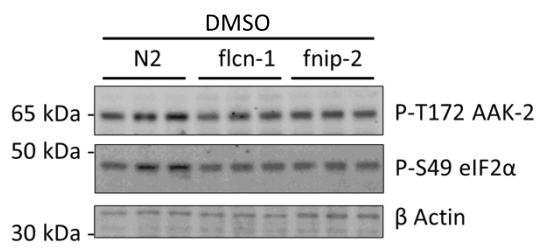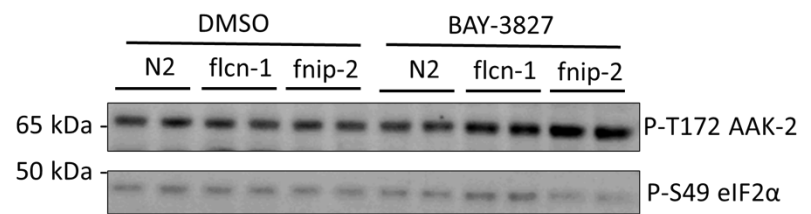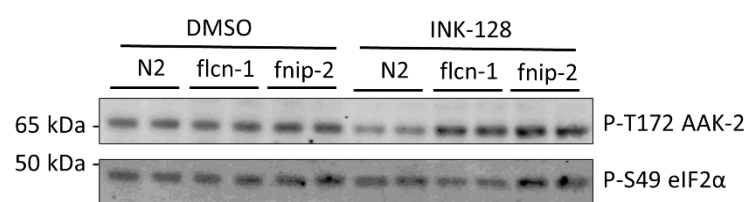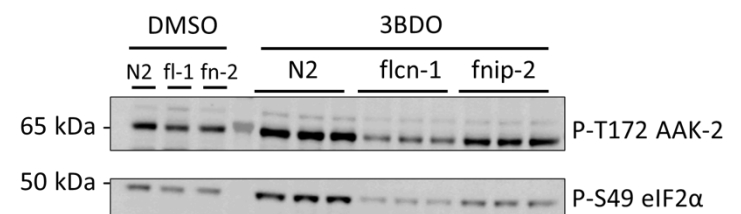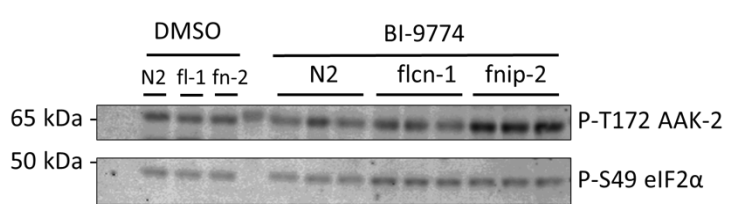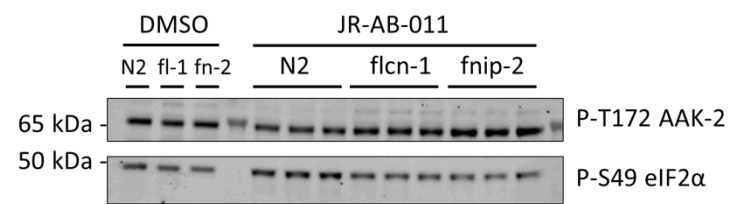

Supplement: Supplementary file 3 — Additional file 3. Western blots of mTOR and AMPK activity. Changes in AMPK and mTOR signalling were monitored using antibodies against P-T172 AMPK and P-S49 eIF2α respectively using protein lysates of N2, flcn-1 and fnip-2 worms treated with the indicated drugs at the same concentrations used for behavioural study. Blots show technical replicates representative of (n = 5), each condition was monitored with two blots, one shown here. Western blotting was performed as described in additional methods (Additional File 6). β Actin was used as loading control. [file 12915_2025_2368_MOESM3_ESM.pdf]

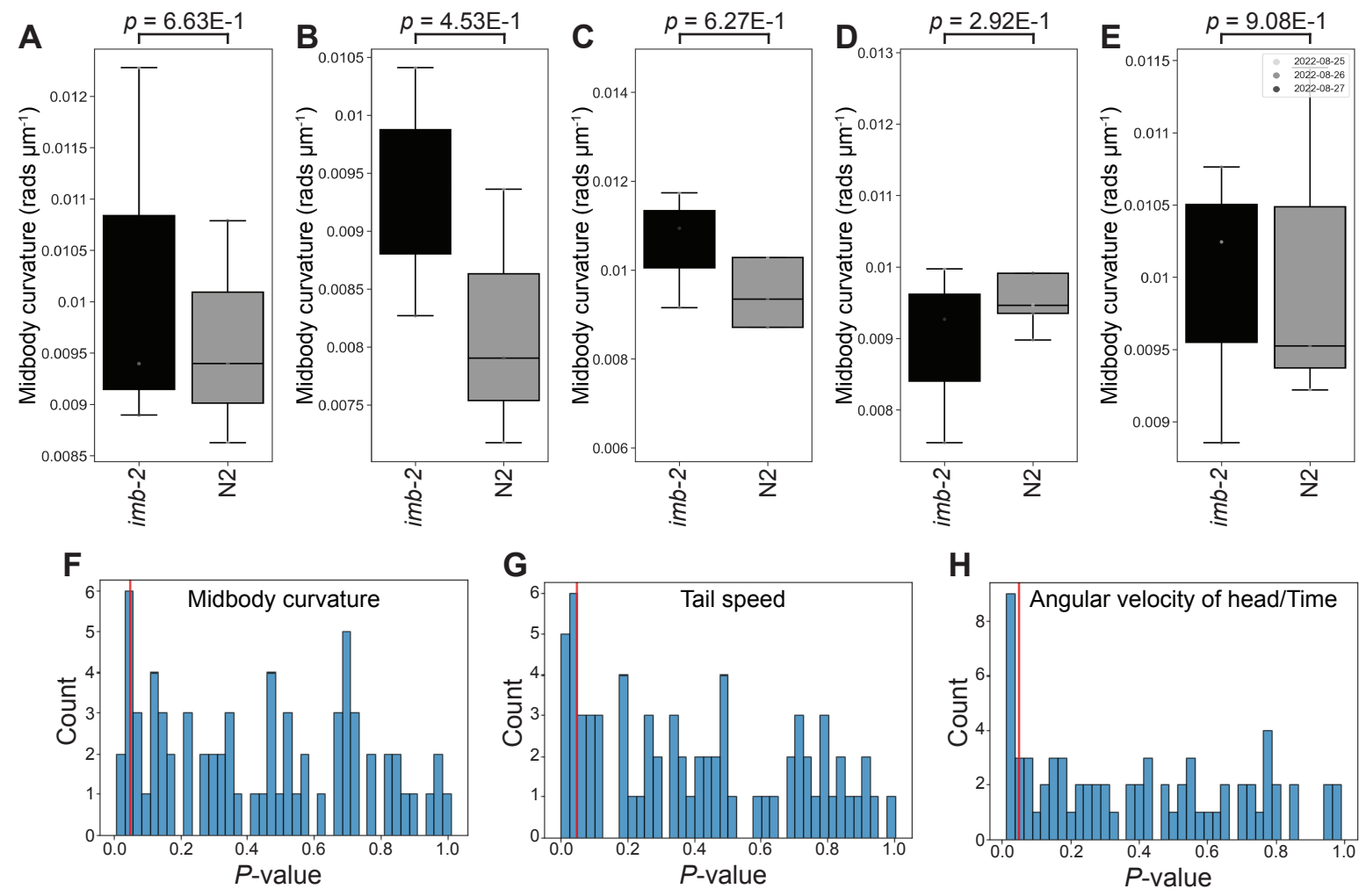

Supplement: Supplementary file 4 — Additional file 4. TNPO2 patient avatar phenotypes cannot be reliably detected with a low number of well replicates. (A-E) Sub-sampled boxplots of the same behavioural feature (midbody curvature) previously detected as being statistically significant between imb-2(syb6372) and wild-type when using a large number of replicates (n > 300, strain specific gene card). Individual plots show randomly sampled data points (n = 3) from the same complete dataset. p-values are for comparison of imb-2[D157N] mutant to N2 using Student’s t-test, correcting for multiple comparisons using the Benjamini- Yekutieli method. (F–H) Histograms of 3 key behavioural features (shown in the strain specific gene card) showing the calculated p-value of each feature when sub-sampling the overall dataset to achieve n = 3 replicates (using same methods as for the individual boxplots). Red line shows p = 0.05, and is considered statistically significant. When looking at a small number of samples (mimicking what may be typically collected for a low replicate screen across a large number of compounds), the behavioural phenotype of the disease model mutant cannot be reliably distinguished from wild-type. [file 12915_2025_2368_MOESM4_ESM.pdf]

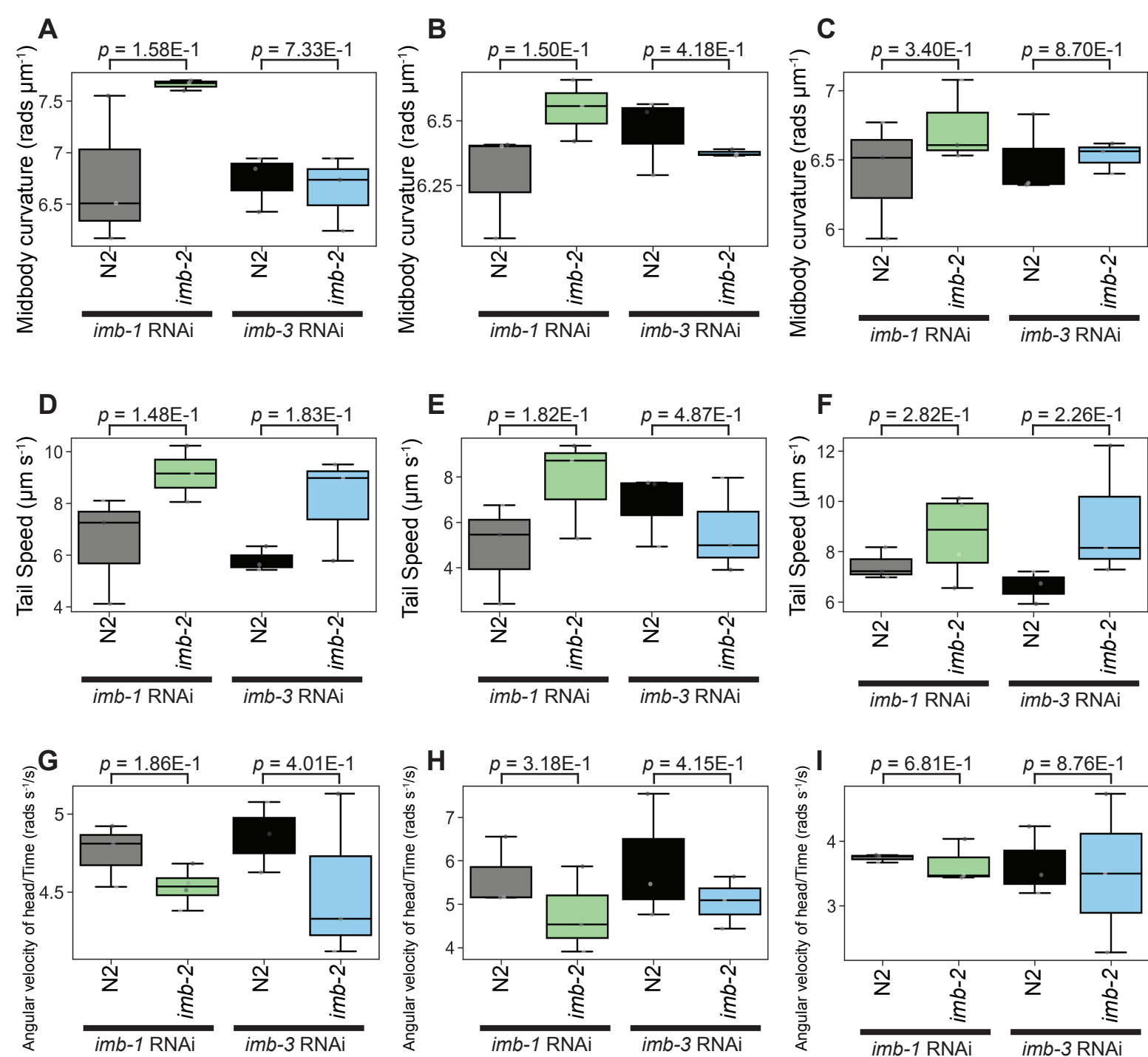

Supplement: Supplementary file 5 — Additional file 5. Targeted sensitisation of TNPO2 patient avatar does not result in a reliable phenotype when using a low number of well replicates. (A-I) Key behavioural phenotype of imb-2(syb6372) and wild-type worms following the RNAi-mediated silencing of imb-1 (left pair of boxes) or imb-3 (right pair of boxes). Individual plots show the same feature (repeated 3 times per row) for randomly sampled points (n = 3) from the complete dataset (shown in Fig. 5). p-values are for comparison of imb-2[D157N] mutant to N2 using Student’s t-test, correcting for multiple comparisons using the Benjamini-Yekutieli method. As in Additional File 4, when looking at a small number of samples the behavioural phenotype of the mutant cannot be reliably distinguished from wild-type. [file 12915_2025_2368_MOESM5_ESM.pdf]
