# Supplementary material for: High-throughput behavioural phenotyping of 25 C. elegans disease models including patient-specific mutations
Source: BMC Biol. 2025 Sep 26;23:281. doi: 10.1186/s12915-025-02368-8 (PMC12465487; doi:10.1186/s12915-025-02368-8)
Supplement: Supplementary file 6 — Additional file 6. [file 12915_2025_2368_MOESM6_ESM.pdf]

Additional Methods

Antibodies

Phospho-AMPKα (Thr172) (40H9) Rabbit mAb #2535, Cell Signaling Technology
Phospho-eIF2α (Ser51) (119A11) Rabbit mAb #3597, Cell Signaling Technology
Beta Actin antibody 66009-1-Ig Clone 2D4H5, Proteintech
Goat anti-Mouse IgG Alexa Fluor Plus 800, Invitrogen A32730,
Goat anti-Rabbit IgG Alexa Fluor Plus 680, Invitrogen A32734

Buffers

MOPS running buffer
Invitrogen™ NuPAGE™ MOPS SDS Running Buffer (20X)
Tris-Glycine transfer buffer
25mM Tris, 192mM glycine
TBST
10mM Tris, pH8, 0.15M NaCl, 0.5% Tween20 (v/v)
4x SLB
0.089 M Tris pH6.8, 13% (v/v) glycerol, 2.7% (v/v) SDS, 7% (v/v) beta mercaptoethanol, 1% (v/v) bromophenol blue)
2X Lysis Buffer
100 mM Hepes pH7.4, 100 mM NaF, 10 mM NaPP, 2 mM EDTA, 20% (v/v) glycerol and 2% Triton X-100 (v/v), 2 mM DTT, 8 µg/ml trypsin inhibitor, 0.2 mM PMSF,
Additionally, 1 PhosSTOP™ tablet and 1 cOmplete™, Mini, EDTA-free Protease Inhibitor Cocktail tablet per 10ml of buffer.

Protein extraction

Packed worm pellets were resuspended in 2x lysis buffer matching the volume of the pellet 1:1 Worm lysate mixes were transferred to 1.5ml tubes with metal beads and put in a bead mill homogenizer at 4 °C. Samples were homogenised at the highest speed for 4 min. Samples were spun down at 19,000 x g for 20 min at 4 °C to clarify the protein extract, this supernatant was used for western blotting.

Western Blotting

15µg of lysate supernatant per sample was used and boiled with 1x SLB for 3 minutes. SDS-PAGE was then carried out using a 4-12% Gradient Polyacrilamide gel Bis-Tris 1mm (Invitrogen NP0323BOX) in MOPS buffer at 180 volts constant voltage for 45 minutes, protein ladder used was PageRuler™ (ThermoScientific 26617). Protein transfer into a PVDF membrane was performed in Tris-Glycine transfer buffer at 4˚C and 40V constant voltage overnight (16h), using a Bio-Rad PROTEAN® Trans-Blot® Module and using it as described in the manufacturer's protocol for overnight transfer (4˚C and 40V).
Membranes were blocked using 4% (w/v) Skim milk powder in Tris Buffered Saline Tween (TBST) for 1 hour then washed once in TBST for 5 minutes, TBST buffer was used to wash after each following step three times for 5 minutes each. Primary antibodies were all used at 1/1000 dilution, 4% BSA (w/v) in TBST. Membranes undergo 16 hours of incubation at 4˚C with primary antibodies. After washing, fluorescently tagged secondary antibodies were used at 1/5000 dilution, 4% (w/v) BSA in TBST, membranes were incubated in them for 1 hour at room temperature. After washing, membranes were scanned using LICOR Odyssey CLx and fluorescence was measured at 680nm and 800nm.
